# Incident disease associations with mosaic chromosomal alterations on autosomes, X and Y chromosomes: insights from a phenome-wide association study in the UK Biobank

**DOI:** 10.1186/s13578-021-00651-z

**Published:** 2021-07-23

**Authors:** Shu-Hong Lin, Derek W. Brown, Brandon Rose, Felix Day, Olivia W. Lee, Sairah M. Khan, Jada Hislop, Stephen J. Chanock, John R. B. Perry, Mitchell J. Machiela

**Affiliations:** 1 Division of Cancer Epidemiology and Genetics, National Cancer Institute, 9609 Medical Center Drive, Rockville, MD 20892 USA; 2 University of Miami Miller School of Medicine, Miami, FL 33136 USA; 3 MRC Epidemiology Unit, University of Cambridge School of Clinical Medicine, Cambridge, UK

**Keywords:** Mosaic loss of Y, Mosaic chromosomal alterations, Phenome-wide association study, PheWAS, UK Biobank

## Abstract

**Background:**

Mosaic chromosomal alterations (mCAs) are large chromosomal gains, losses and copy-neutral losses of heterozygosity (LOH) in peripheral leukocytes. While many individuals with detectable mCAs have no notable adverse outcomes, mCA-associated gene dosage alterations as well as clonal expansion of mutated leukocyte clones could increase susceptibility to disease.

**Results:**

We performed a phenome-wide association study (PheWAS) using existing data from 482,396 UK Biobank (UKBB) participants to investigate potential associations between mCAs and incident disease. Of the 1290 ICD codes we examined, our adjusted analysis identified a total of 50 incident disease outcomes associated with mCAs at PheWAS significance levels. We observed striking differences in the diseases associated with each type of alteration, with autosomal mCAs most associated with increased hematologic malignancies, incident infections and possibly cancer therapy-related conditions. Alterations of chromosome X were associated with increased lymphoid leukemia risk and, mCAs of chromosome Y were linked to potential reduced metabolic disease risk.

**Conclusions:**

Our findings demonstrate that a wide range of diseases are potential sequelae of mCAs and highlight the critical importance of careful covariate adjustment in mCA disease association studies.

**Supplementary Information:**

The online version contains supplementary material available at 10.1186/s13578-021-00651-z.

## Introduction

Cells accumulate somatic mutations during normal growth and cellular division [36, 48], despite the presence of cellular mechanisms to prevent and repair genomic damage [19]. In many cases, acquired somatic mutations are not compatible with cellular survival resulting in apoptosis [9]. However, some mutations can evade repair mechanisms and are tolerated by cells [40, 48]. These surviving mutations could provide competitive advantage over normal cells lacking these somatic mutations. Clonal expansion of these cells result in genetic mosaicism, the presence of a clonal subset of cells with a mutation that differs from normal germline DNA [31, 43]. As mCAs generally cover an expansive genomic footprint (often more than 50 kilobases), these events offer opportunities to investigate future health outcomes that could arise due to a non-trivial fraction of the genome impacted by a somatic alteration [53].

Peripheral leukocytes are perhaps the most well-studied tissue for somatic mutations as blood-derived DNA is easy to obtain for large populations of individuals [14, 20, 23, 28, 29, 34, 42, 46]. Clonal hematopoiesis (CH) is an age-related process in which a hematopoietic stem cell which has acquired a somatic mutation, passes that mutation onto daughter cells. The chance of observing these subclones carrying genomic alterations increases with increasing age. These daughter cells eventually form a clonal subpopulation of cells with the somatic mutations offering potential cellular survival advantages. Genomic alterations can range in size from a single base pair change (e.g., somatic SNVs) [16, 21, 22], to very large structural mosaic chromosomal alterations (mCAs) [20, 23, 28, 29, 34, 37, 42, 46]. mCAs can be divided into different categories according to the location and span of these events, i.e. telomeric, centromeric, interstitial, and whole chromosome. Additionally, copy number changes can be used to classify these alterations as mosaic chromosomal gains, mosaic chromosomal losses, and mosaic chromosomal losses of heterozygosity (LOH) [34].

Mosaic loss of the Y chromosome (mLOY) is the most frequently occurring mCA in males [12, 20, 46, 50, 56]. The frequency of mLOY increases with age, with < 2.5% of men below the age of 40 having detectable mLOY and estimated frequencies rapidly increasing in excess of 40% by 70 years of age [46, 56]. A number of studies have investigated the impact of mLOY on future health outcomes and noted possible associations with risk of solid tumors [13, 25, 26, 56], Alzheimer's disease [7], cardiovascular disease [18] and other chronic health outcomes [11, 17]. However, the level of evidence for these associations varies by study and some lack adjustment for key confounders like smoking patterns. Likewise, some studies of autosomal mosaicism have reported associations with risk of cancer; particularly hematologic cancers [20, 28], diabetes [1], and infectious disease [54]. Current investigation of phenotypes associated with mosaic loss of the X chromosome (mLOX) in females is limited [33]. mLOX is not observed in males since it is critical male cells carry at least one copy of the X chromosome [55].

Herein, we perform a phenome-wide association study (PheWAS) of 1290 first occurrence of incident health conditions (both self-reported and a subset clinically verified) among 482,396 UK Biobank (UKBB) participants [44]. Our aim is to systematically scan for associations between risk of disease and presence of mCAs. Our approach has been to perform robust statistical adjustment, require stringent significance levels and carry out additional sensitivity analyses to identify a high-confidence set of incident disease associations to be prioritized in future studies of mCAs.

## Methods

### Study population

UK Biobank sample collection and processing protocols are previously described [8]. In brief, UK Biobank enrolled approximately half a million participants, aged 37 to 73, from 22 assessment centers across the UK from 2006 to 2010. Participants provided informed consent for collection of medical histories using touch-screen questionnaires, participation in nurse-led interviews, and blood sample collection at enrollment. 4.5 mL of blood samples were collected in anticoagulant tubes containing acid citrate dextrose, EDTA, or lithium-heparin as well as silica-containing tubes for fast clotting. Upon arrival at the central processing laboratory, blood was aliquoted and cryopreserved at − 80 °C or in liquid nitrogen within 24 h.

### Genotyping and detection of mosaicism

DNA extracted from the blood samples was genotyped on the Applied Biosystems UK BiLEVE or UK Biobank Axiom array. After removing individuals with sex discordance or whose DNA failed QC during genotyping, 482,396 individuals with genotype and phenotype data were included for analyses. We used previously generated data on copy number variation and corresponding cellular fraction [28, 29]. Additional steps designed for detecting mosaic Y loss events were performed as described previously [46]. In brief, log transformed intensities from UK Biobank genotyping arrays were used to yield log_2_ R ratio and B-allele frequency for each SNP, and the Eagle2 software [27, 30] was employed to phase SNPs. After phasing, a hidden Markov model was employed to detect allelic imbalances using additional information from long-range haplotype data. Chromosome Y events were detected using B-allele frequency in two pseudo-autosomal regions (PAR) shared between the Y and X chromosomes, and further examination of log_2_ R ratio across the whole chromosome was employed to determine copy number changes [46].

### Phenotypes

First occurrence of 1764 ICD-10-coded diseases (UK Biobank category 1712) were derived from linkage to primary care, hospital admission, cancer registry, death register data as well as self-report disease history obtained at enrollment. We further combined these data with the most up-to-date inpatient data (UK Biobank fields 41270 and 41280) as well as cancer registry data (UK Biobank fields 40005 and 40006). For the main PheWAS [2, 6] analysis, we first investigated incident cases defined by diagnoses occurring after enrollment; for simplicity in performing the PheWAS, individuals with diseases diagnosed before enrollment were coded as controls. To ensure that this control coding scheme did not bias reported associations, sensitivity analyses by cancer status and additional adjustment for ancestry as well as Mendelian randomization were performed. Further exploratory analyses focused on prevalent disease and medication codes to more comprehensively examine associations with mCAs.

### Medications

The UK Biobank has 6745 unique medication codes (UK Biobank category 20003) derived from the Read Codes, Clinical Terms Version 3 (CTV3). As prior publications curating UK Biobank medications were incomplete [51] or based on text interpreters (Categorising UK Biobank Self-Reported Medication Data using Text Matching [3]), we traced the origins of each UK Biobank medication code back to the original CTV3 Read Codes to assign standardized active ingredients to each code. All UK Biobank medication codes were converted with high fidelity (100%) to 6197 unique CTV3 Read Codes along with hierarchy-path information to 1073 single ingredients and assigned Unified Medical Language System (UMLS) RxNorm codes using the “RxNormR” package in R. RxNorm codes provided connections to other medication classification systems including ATC (WHO), DrugBank (University of Alberta and the Metabolomics Innovation Centre) and MeSH (NLM thesaurus). In some cases, manual edits were required to provide missing ATC codes and exclude codes that did not apply based on CTV3 path information. Finally, PheWAS analysis of mCAs was performed on the reported medications using each of the five levels of ATC codes: chemical substance, chemical subgroup, pharmacological subgroup, therapeutic subgroup, and anatomical main group to identify certain components of medications that may be associated with mCAs.

### Statistical analyses

All statistical analyses were performed in R version 4.0.3 [41]. PheWAS was performed using the “PheWAS” package [2]. Variables adjusted in the PheWAS models include age, age^2^, sex (for analyses other than loss of X or Y chromosomes), and a detailed 25-level smoking variable. The detailed 25-level smoking variable was created as previously described [25] and includes information on smoking intensity, duration and tobacco type. Genetic ancestry proportions were calculated using SNPweights [4], which utilizes SNP weights computed from large reference panels to infer genetic ancestry. The percentage of European, African, and Asian ancestry were computed for each participant, and these percentages were used in adjusted analyses. Phenome-wide significance levels were calculated by Bonferroni correction (0.05/number of diseases tested), in which the number of diseases tested vary by the category of genomic alteration investigated. We set a minimum number of cases for inclusion in analyses of 20 to perform reliable asymptotic association tests. In total, autosomal mCA models with participants of both sexes had 1290 incident diseases with sufficient case numbers, mLOY models with males had 1140 incident diseases, and mLOX models with females included 1128 incident diseases. Manhattan plots for the PheWAS were created with the “ggplot2” package [49].

We utilized the “GLIDE” package to test potential pleiotropy [5] and applied the Steiger filter to remove mLOY-related SNPs which could be subject to reverse causation from either body mass index or high blood sugar. With a false discovery rate threshold of 0.05, 127 out of 156 previously published mLOY-related SNPs [46] were chosen as the mLOY instrument. Mendelian randomization analyses were conducted with the “MendelianRandomization” package [52].

## Results

### Detectable mCAs in the UK Biobank population

A total of 482,396 individuals were examined for mCAs, with a total of 17,113 (3.5%) having at least one detectable autosomal event and a total of 19,632 mosaic chromosomal alterations detected on the autosomes. Of the detected autosomal events, 8185 (41.7%) were copy number neutral, 3718 (18.9%) were losses, 2389 (12.2%) were gains, and 5341 (27.2%) had undetermined copy number state due to challenges with assigning copy number status to events with a low cellular fraction of affected cells (Additional file 1: Fig. S1). In addition to mosaic events of the autosomes, we also identified 43,297 males (19.6%) with mosaic chromosome Y loss (mLOY) and 12,550 females (4.8%) with chromosome X loss (mLOX). There were no cases of mLOX detected in males.

### Observed associations between mosaic chromosomal alterations and incident disease

**Fig. 1:**
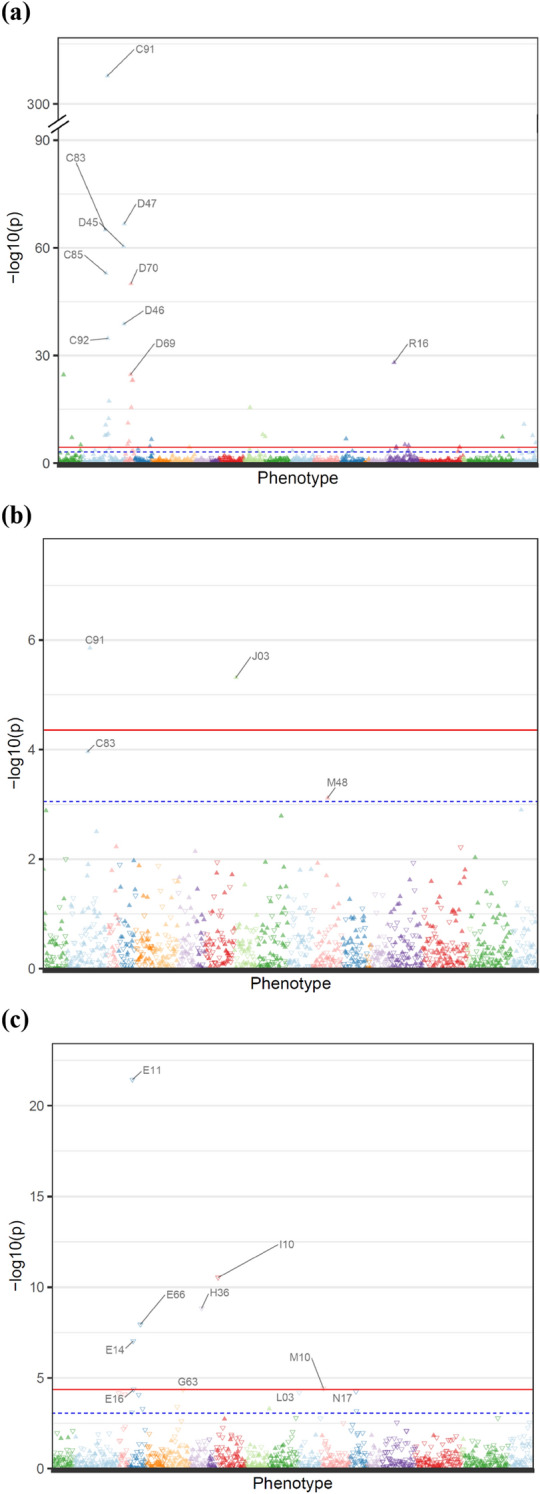
Manhattan plots of incident disease diagnoses and mCA in UKBB organized by disease category/anatomical location. All models are adjusting for age, age^2^, detailed 25-level smoking, and sex (only for autosomal mCA analyses). Upward closed triangles represent diseases with positive associations while downward open triangles represent diseases with negative associations. Colors are used to separate diseases in different ICD-10 phenotype blocks. The red line represents the PheWAS significance level (Bonferroni correction: 0.05/number of diseases tested) and the blue dashed line represents the suggestive threshold (1/number of diseases tested). A maximum of the ten most significant medication codes above the suggestive threshold are labeled. PheWAS results are for: **a** Autosomal mCAs, 1290 diseases tested (PheWAS significance level: 3.88 × 10^–5^, suggestive line: 7.75 × 10^–4^); C91 Lymphoid leukaemia, C92 Myeloid leukaemia, C85 Other and unspecified types of non-Hodgkin's lymphoma, C83 Diffuse non-Hodgkin's lymphoma, D47 Other neoplasms of uncertain or unknown behaviour of lymphoid, haematopoietic and related tissue, D45 Polycythaemia vera, D69 Purpura and other haemorrhagic conditions, D46 Myelodysplastic syndromes, D70 Agranulocytosis, and R16 Hepatomegaly and splenomegaly, not elsewhere classified. **b** mLOX, 1,128 diseases tested (PheWAS significance level: 4.43 × 10^–5^, suggestive line: 8.87 × 10^–4^); C91 Lymphoid leukaemia, C83 Diffuse non-Hodgkin's lymphoma, J03 Acute tonsillitis, and M48 Other spondylopathies. **c** mLOY, 1140 diseases tested (PheWAS significance level: 4.39 × 10^–5^, suggestive line: 8.77 × 10^–4^); E11: Non-insulin-dependent diabetes mellitus, E14: Unspecified diabetes mellitus, E16: Other disorders of pancreatic internal secretion, E66: Obesity, H36: Retinal disorders in diseases classified elsewhere, I10: Essential (primary) hypertension, G63: Polyneuropathy in diseases classified elsewhere, L03: Cellulitis, M10: Gout, and N17: Acute renal failure

We first performed a PheWAS for incident diseases that were captured in the UKBiobank resource (Table 1, Fig. 1a). We observed the strongest association between autosomal mCAs and risk of C91 lymphoid leukemia (OR 23.74, p < 5 × 10^–324^). In addition to lymphoid leukemia, we also observed a strong association with C83 diffuse non-Hodgkin’s lymphoma (OR 4.62, p = 9.64 × 10^–66^). However, lymphoid lineage malignancies were not the only hematologic malignancy associated with autosomal mCAs; diseases of the myeloid lineage were also found to be associated, including D45 polycythaemia vera (OR 12.26, p = 3.37 × 10^–61^), D46 myelodysplastic syndromes (OR 6.19, p = 1.65 × 10^–39^), and C92 myeloid leukemia (both acute C92.0 and chronic C92.1 myeloid leukemia; OR 5.19, p = 1.66 × 10^–35^). This suggests that the association between mCAs and myeloid and lymphoid lineage diseases points towards an early progenitor cell, leading to abnormal growth of distinct sets of blood cells. Additionally, we detected associations which could be partially attributed to cancer progression as well as treatment evidenced by simultaneous or prior diagnoses of neoplasm. Diagnoses included D70 agranulocytosis (OR 2.24, p = 9.73 × 10^–51^), A41 other septicaemia (OR 1.54, p = 2.43 × 10^–25^), D80 immunodeficiency with predominantly antibody defects (OR 5.44, p = 7.27 × 10^–24^), D61 other aplastic anemia (OR 2.24, p = 6.94 × 10^–12^), J18 pneumonia of unspecified organism (OR 1.32, 3.19 × 10^–16^), J90 pleural effusion (OR 1.29, p = 1.06 × 10^–8^), B25 cytomegaloviral disease (OR 3.63, p = 7.78 × 10^–8^), and T86 failure and rejection of transplanted organs and tissues (OR 2.60, p = 3.39 × 10^–5^). When stratifying by cancer diagnoses prior to these diseases, most effect estimates were higher in magnitude in people with a prior cancer diagnosis (Additional file 1: Table S1); this is despite much larger variation in these strata due to smaller sample sizes. Sensitivity analyses with additional adjustment of genetic ancestry resulted in the same statistically significant findings in autosomal mCAs, except for C93 monocytic leukemia (Additional file 1: Table S2). In this case, the model of monocytic leukemia failed to converge suggesting uneven ancestral distribution of monocytic leukemia among UK Biobank participants. PheWAS on each autosome found associations with a very similar set of diseases and demonstrated that blood organ malignancies were often associated with mCAs on multiple autosomes ( Additional file 1: Tables S3, S4).

**Table 1:** Statistically significant associations for mCAs and incident disease associations.

mCA	Disease	N	Percent with preceding cancer	Odds ratio [95% Confidence Interval]	P-value
Autosome	C91 Lymphoid leukaemia	616	35.47	23.74 [20.21–27.88]	< 5 × 10^–324^
Autosome	D47 Other neoplasms of uncertain or unknown behaviour of lymphoid, haematopoietic and related tissue	729	37.5	5.29 [4.38–6.39]	1.97 × 10^–67^
Autosome	C83 Diffuse non-Hodgkin's lymphoma	903	50.67	4.62 [3.88–5.5]	9.64 × 10^–66^
Autosome	D45 Polycythaemia vera	201	21.47	12.26 [9.1–16.51]	3.37 × 10^–61^
Autosome	C85 Other and unspecified types of non-Hodgkin's lymphoma	859	59.02	4.28 [3.56–5.15]	1.27 × 10^–53^
Autosome	D70 Agranulocytosis	4637	72.65	2.24 [2.02–2.49]	9.73 × 10^–51^
Autosome	D46 Myelodysplastic syndromes	302	47.86	6.19 [4.72–8.13]	1.65 × 10^–39^
Autosome	C92 Myeloid leukaemia	381	47.22	5.19 [4–6.73]	1.66 × 10^–35^
Autosome	R16 Hepatomegaly and splenomegaly, not elsewhere classified	611	36.33	3.78 [2.99–4.77]	9.44 × 10^–29^
Autosome	D69 Purpura and other haemorrhagic conditions	3090	38.04	2.01 [1.76–2.29]	2.02 × 10^–25^
Autosome	A41 Other septicaemia	10,144	52.41	1.54 [1.42–1.67]	2.43 × 10^–25^
Autosome	D80 Immunodeficiency with predominantly antibody defects	248	42.63	5.44 [3.91–7.56]	7.27 × 10^–24^
Autosome	D75 Other diseases of blood and blood-forming organs	1059	25.52	2.77 [2.27–3.38]	9.33 × 10^–24^
Autosome	C95 Leukaemia of unspecified cell type	50	76.92	13.37 [7.43–24.06]	4.92 × 10^–18^
Autosome	D72 Other disorders of white blood cells	794	27.25	2.66 [2.1–3.36]	2.97 × 10^–16^
Autosome	J18 Pneumonia, organism unspecified	17,638	29.62	1.32 [1.24–1.41]	3.19 × 10^–16^
Autosome	C94 Other leukaemias of specified cell type	32	70.59	14.93 [7.2–30.96]	3.73 × 10^–13^
Autosome	D61 Other aplastic anaemias	879	46.57	2.24 [1.78–2.82]	6.94 × 10^–12^
Autosome	Z51 Other medical care	23,428	63.39	1.24 [1.16–1.32]	1.38 × 10^–11^
Autosome	C84 Peripheral and cutaneous T-cell lymphomas	154	41.08	4.37 [2.83–6.72]	2.27 × 10^–11^
Autosome	C93 Monocytic leukaemia	51	80.77	6.61 [3.5–12.47]	5.89 × 10^–9^
Autosome	J90 Pleural effusion, not elsewhere classified	10,097	42.9	1.29 [1.18–1.41]	1.06 × 10^–8^
Autosome	C88 Malignant immunoproliferative diseases	121	69.7	4.17 [2.55–6.83]	1.43 × 10^–8^
Autosome	C82 Follicular [nodular] non-Hodgkin's lymphoma	347	37.96	2.71 [1.91–3.83]	1.94 × 10^–8^
Autosome	Z85 Personal history of malignant neoplasm	20,384	88.65	1.21 [1.13–1.29]	1.98 × 10^–8^
Autosome	J98 Other respiratory disorders	12,465	29.57	1.26 [1.16–1.37]	4.06 × 10^–8^
Autosome	Y43 Primarily systemic agents	2494	93.78	1.58 [1.34–1.86]	5.24 × 10^–8^
Autosome	B25 Cytomegaloviral disease	171	51.13	3.63 [2.27–5.81]	7.78 × 10^–8^
Autosome	N17 Acute renal failure	14,465	42.7	1.22 [1.13–1.32]	1.61 × 10^–7^
Autosome	E87 Other disorders of fluid, electrolyte and acid–base balance	15,028	38.77	1.22 [1.13–1.31]	2.40 × 10^–7^
Autosome	D64 Other anaemias	16,970	30.46	1.2 [1.11–1.29]	9.06 × 10^–7^
Autosome	Z94 Transplanted organ and tissue status	724	44.28	1.99 [1.5–2.65]	1.94 × 10^–6^
Autosome	D59 Acquired haemolytic anaemia	177	38.52	2.96 [1.85–4.73]	5.70 × 10^–6^
Autosome	R50 Fever of unknown origin	3504	48.62	1.4 [1.21–1.63]	6.79 × 10^–6^
Autosome	B96 Other bacterial agents as the cause of diseases classified to other chapters	9917	33.45	1.23 [1.12–1.35]	8.21 × 10^–6^
Autosome	R60 Oedema, not elsewhere classified	2075	42.78	1.49 [1.25–1.78]	1.11 × 10^–5^
Autosome	E83 Disorders of mineral metabolism	5293	43.15	1.3 [1.15–1.47]	2.23 × 10^–5^
Autosome	R22 Localised swelling, mass and lump of skin and subcutaneous tissue	1175	28.16	1.66 [1.31–2.11]	2.82 × 10^–5^
Autosome	G72 Other myopathies	258	28.99	2.45 [1.6–3.74]	3.29 × 10^–5^
Autosome	T86 Failure and rejection of transplanted organs and tissues	232	49.76	2.6 [1.65–4.08]	3.39 × 10^–5^
mLOX	C91 Lymphoid leukaemia	236	35.47	2.45 [1.7–3.53]	1.40 × 10^–6^
mLOX	J03 Acute tonsillitis	1167	4.97	1.78 [1.39–2.27]	4.85 × 10^–6^
mLOY	E11 Non-insulin-dependent diabetes mellitus	14,110	21.37	0.8 [0.77–0.84]	3.67 × 10^–22^
mLOY	I10 Essential (primary) hypertension	46,694	20.37	0.91 [0.89–0.94]	2.91 × 10^–11^
mLOY	H36 Retinal disorders in diseases classified elsewhere	1359	20.69	0.65 [0.56–0.75]	1.46 × 10^–9^
mLOY	E66 Obesity	12,647	22.40	0.87 [0.83–0.91]	1.12 × 10^–8^
mLOY	E14 Unspecified diabetes mellitus	1642	11.07	0.7 [0.61–0.8]	9.34 × 10^–8^
mLOY	M10 Gout	5601	14.55	0.87 [0.81–0.93]	3.74 × 10^–5^
mLOY	E16 Other disorders of pancreatic internal secretion	1641	30.03	0.78 [0.69–0.88]	4.28 × 10^–5^
mLOY	G63 Polyneuropathy in diseases classified elsewhere	646	29.78	0.65 [0.53–0.8]	4.29 × 10^–5^

Only PheWAS significant associations are reported (P < 3.88 × 10^–5^ for autosomal, P < 4.43 × 10^–5^ for mLOX, and P < 4.39 × 10^–5^ for mLOY). All PheWAS associations are adjusted for age, age^2^, smoking, and sex (only for autosomal mCA analyses). We define percent with preceding cancer as the percentage of individuals who have been diagnosed with any cancer prior to the diagnosis of that disease

For mLOX events in women, we found an increased risk of C91 lymphoid leukemia (OR 2.45, p = 1.40 × 10^–6^) as well as an unrelated outcome, J03 acute tonsillitis (OR 1.78, p = 4.85 × 10^–6^) (Table 1, Fig. 1b). mLOY had an entirely different spectrum of disease associations from mLOX and autosomal mCAs. Not only was there an absence of an association with blood organ and tissue-related diseases, we observed protective associations between mLOY and a series of metabolic conditions: E11 type II diabetes (OR 0.80, p = 3.67 × 10^–22^), M10 gout (OR 0.87, p = 3.74 × 10^–5^), E66 obesity (OR 0.87, p = 1.12 × 10^–8^), H36 retinal disorders (OR 0.65, p = 1.46 × 10^–9^), and I10 primary hypertension (OR 0.91, p = 2.91 × 10^–11^) (Table 1, Fig. 1c).

### Examining the potential protective effects of mLOY with incident metabolic diseases

To investigate connections with a range of metabolic diseases, we performed additional analyses to better examine the impact of established common underlying confounders (e.g., prior cancer history or BMI). A sensitivity analysis in a subset excluding individuals with prior cancer history, did not find evidence that prior history of cancer is a confounder in our study (Additional file 1: Table S5). However, adjustment for BMI completely attenuated most of the negative associations between mLOY and metabolic diseases. The retinal disorders (OR 0.65, p = 6.36 × 10^–8^) and primary hypertension (OR 0.94, p = 1.35 × 10^–6^) remained negatively associated after BMI adjustment, albeit with attenuated effect sizes (Additional file 1: Table S6). We examined potential underlying causes of retinal diseases by investigating other comorbidities in the group of patients with incident retinal disease (n = 3752). Among individuals with retinal disorders, 3408 (90.8%) were diagnosed with non-insulin dependent diabetes, 3103 (82.7%) had primary hypertension, and 3073 (81.9%) had unspecified diabetes. In total, 98.8% of all patients suffering retinal disorders had at least one of the three aforementioned diseases suggesting the association of mLOY with retinal disease may be a result of these underlying conditions that predispose to retinal disease.

To further examine the observed protective association between Y loss and the two traits that remained significant after BMI adjustment (hypertension and retinal diseases), Mendelian randomization (MR) was performed on previously-reported germline variants associated with mLOY [46]. We used Steiger filter to remove SNPs which could be associated with mLOY through effects on hypertension or abnormal blood sugar (see Methods). Table 2 shows the effect estimates from the observational study and MR (IVW method). The Mendelian Randomization (MR) analyses for retinal diseases remained statistically significant, and the MR effect estimate was in the same direction suggesting that mLOY could be an independent protective factor for retinal disease, although further investigation is needed.

**Table 2:** Comparison of effect estimates between observational associations and Mendelian randomization (MR) estimates from inverse variance weighted (IVW) method.

	Observation		MR IVW	
Trait	Odds ratio [95% confidence interval]	P-value	Odds ratio [95% confidence interval]	P-value
Retinal diseases	0.67 [0.58–0.77]	6.06 × 10^–8^	0.77 [0.68–0.87]	4.49 × 10^–5^
Hypertension	0.92 [0.89–0.96]	5.11 × 10^–10^	0.99 [0.97–1.02]	0.175

Observational associations adjust for age, age^2^, and 25-level smoking. MR estimates use selected variants previously identified to be associated with mLOY susceptibility (Thompson et al. [46])

### Exploratory analyses of mosaicism and prevalent disease

We also tested for associations between the three types of detectable mosaicism (autosomal, LOX, and LOY) and prevalent disease. We observed 39 phenome-wide significant (Bonferroni corrected p < 3.88 × 10^–5^) disease associations with autosomal mosaic events, 62 (p < 4.43 × 10^–5^) disease associations with mLOX, and 149 phenome-wide significant (p < 4.39 × 10^–5^) disease associations with mLOY (Additional file 1: Table S7, Fig S2). Caution should be exercised in interpretation of these prevalent disease associations due to unknown timing of both the primary diagnoses and detectible mosaicism as well as the fact that important confounders were measured at study enrollment and might not reflect the status of participants when diseases and mosaicism first occurred.

We observed several prevalent diseases that were associated with mosaic events across autosomes and both sex chromosomes. The most frequently observed disease associations were in diseases related to prevalent cardiovascular or metabolic disease. A number of ageing-related conditions, like coxarthrosis, other arthrosis (M19 other arthrosis includes M19.0 primary arthrosis, M19.1 posttraumatic arthrosis, M19.2 other secondary arthrosis, and M19.8 other specified arthrosis, and M19.9 unspecified arthrosis), osteoporosis without fracture, and spondylosis, were also associated with all forms of mosaicism. Other observed prevalent disease associations included contraceptive management, vasomotor and allergic rhinitis.

Prevalent conditions associated with both autosomal mosaicism and mLOY in men included a history of malignant neoplasm, hyperplasia or neoplasm of prostate, atherosclerosis, and inguinal hernia. In women, autosomal mCAs and mLOX were both negatively associated with prevalent excessive, frequent and irregular menstruation.

A number of conditions shared between mLOY and mLOX were not associated with autosomal mCAs. Diseases of the respiratory and digestive systems were the most frequently found to be positively associated with sex chromosome loss in both sexes. We also observed associations for sex chromosome loss with diseases in the urinary system, including chronic renal failure and unspecified hematuria.

### Observed associations between mosaicism and medication ATC codes

**Fig. 2:**
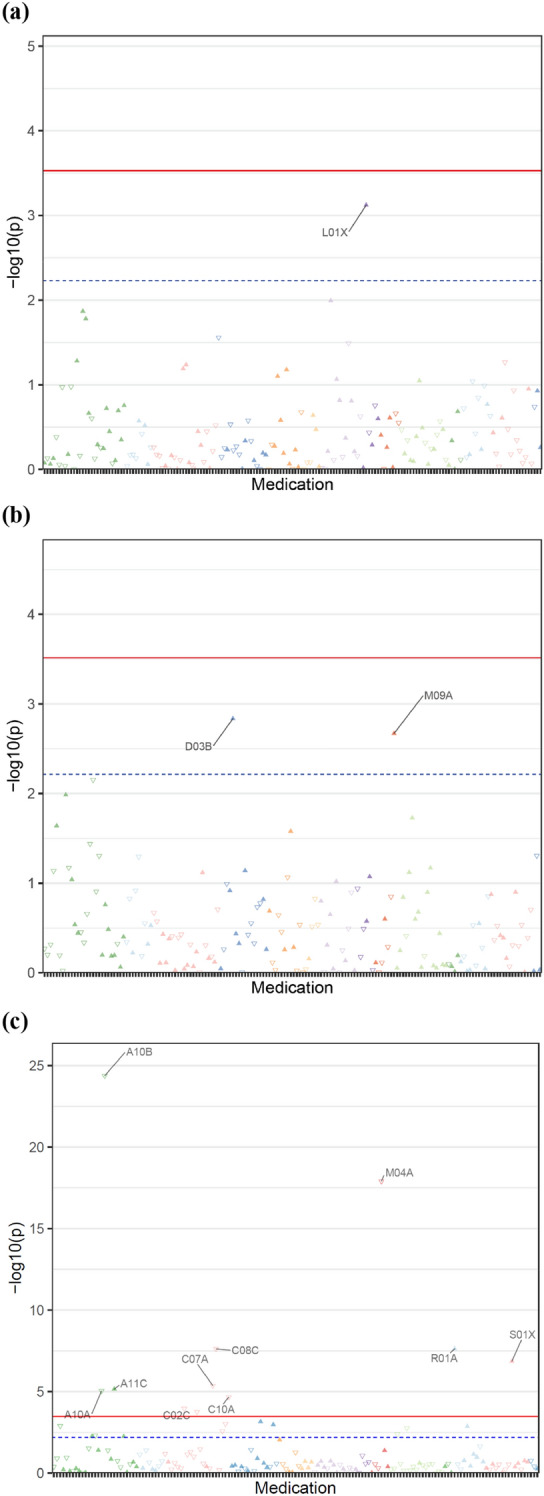
Manhattan plots of ATC medication codes (level 3) and mCAs in the UK Biobank. All associations are adjusted for age, age^2^, detailed 25-level smoking, and sex (only for autosomal mCA analyses). Colors are used to separate medications in different ATC level 1 categories. The red line represents the Bonferroni-corrected, PheWAS significance level (0.05/number of medication codes tested). The blue dashed line represents the minimum, suggestive threshold (1/number of medication codes tested). A maximum of the ten most significant medication codes above the suggestive threshold are labeled. PheWAS associations are reported for: **a** Autosomal mCAs, 169 medication codes tested (PheWAS significance: p < 2.96 × 10^–4^, suggestive threshold: 5.92 × 10^–3^); L01X Other antineoplastic agents. **b** mLOX, 164 medication codes tested (PheWAS significance level p < 3.05 × 10^–4^, suggestive threshold: 6.10 × 10^–3^); D03B Enzymes for treatment of wounds and ulcers and M09A Other drugs for disorders of the musculo-skeletal system. **c** mLOY, 153 medication codes tested (PheWAS significance level p < 3.27 × 10^–4^, suggestive threshold: 6.54 × 10^–3^); A10A Insulins and analogues, A10B Blood glucose lowering drugs, excluding insulin, A11C Vitamin A and D, including combinations of the two, C02C Antiandrenergic agents, peripherally acting, C07A Beta blocking agents, C08C Selective calcium channel blockers with mainly vascular effects, C10A Lipid modifying agents, plain, M04A Antigout preparations, R01A Decongestants and other nasal preparations for topical use, and S01X Other opththalmologicals

UK Biobank participant medications were converted to WHO Anatomic Therapeutic Chemical (ATC) codes and screened for associations for mCAs at different ATC code levels. Figure 2 and Additional file 1: Table S8 demonstrate that while we did not identify statistically significant associations between ATC level 3 medications and autosomal mCAs (p < 3.27 × 10^–4^) or mLOX (p < 3.27 × 10^–4^), mLOY (p < 3.27 × 10^–4^) were shown to be negatively associated with blood glucose lowering drugs (OR 0.73, p = 4.24 × 10^–25^), insulins and analogues (OR 0.78, p = 9.18 × 10^–6^), as well as calcium channel blockers (OR 0.89, p = 2.42 × 10^–8^), beta blockers (OR 0.91, p = 4.29 × 10^–6^), and antigout preparations (OR 0.68, p = 1.25 × 10^–18^). These results are consistent with the protective associations with incident diseases.

## Discussion

In this study, we examined plausible associations between detectible mCAs and first occurrence of a range of diseases in a large, well-characterized population of approximately 500,000 individuals. We utilized existing high-confidence mosaic alteration calls [28, 29, 46] and created a comprehensive aggregation of first occurrence of diseases by integrating primary care, inpatient data, death registry, cancer registry as well as self-reported disease history obtained through interview at study enrollment. Our analyses identified mCA-disease associations, many of which were associated with specific types of mCA, suggesting distinct mechanisms or perturbations of key pathways in their pathogenesis. Examination of potential confounders using sensitivity analysis and adjustment demonstrated the critical importance of careful covariate adjustment in CH-disease association studies to avoid reporting spurious associations.

We observed robust evidence for known associations between autosomal mCAs and hematologic cancer risk. We observed the strongest associations with lymphoid malignancies, and also noted substantial evidence for myeloid malignancies. This suggests that autosomal mCAs predispose to cancer risk in both the lymphoid and myeloid compartments. Consistent with prior reports, we observed evidence for potential associations between autosomal mCAs and various blood organ-related diseases as well as infections possibly linked to cancer treatment [54]. These findings indicate that clonal expansion of aberrant mCA clones can have an overall effect on select blood components that manifest in distinct detectable and diagnosable diseases. Such clonal expansion of autosomal mCAs could promote blood tissue disease risk by reducing stem cell diversity, impacting blood cell differentiation and altering leukocyte function due to copy-number or loss of heterozygosity (LOH) induced changes in gene regulation and expression. Similar to the autosomes, mLOX was associated with increased risk of lymphoid leukemia suggesting mLOX events may also predispose to hematologic cancer risk. Likewise, we observed an association between mLOX and acute tonsillitis indicating mLOX could play a role in infection or immune response as well.

The spectrum of incident diseases associated with mLOY differed substantially from autosomal mCAs and mLOX. As mLOY is the most commonly observed mCA in men, the UK Biobank male population had increased power for detecting incident disease associations with mLOY. Despite this improved power for detecting associations, we observed a paucity of evidence for incident disease associations with mLOY. It is notable that previously published mLOY associations were not confirmed after Bonferroni correction (p < 4.39 × 10^–5^), such as solid tumor risk [13, 26], Alzheimer’s disease [7], major cardiovascular events [18], age-related macular degeneration [17], autoimmune thyroiditis [39], primary biliary cirrhosis [24], testicular germ cell tumor [32], and abdominal aortic aneurysm [45]. While these findings could indicate limited evidence for associations with mLOY and incidence of these diseases in the UK Biobank male population, we noted the multiple testing correction threshold for significance was stringent and that the generally healthy participant pool in the UK Biobank male participants [15] may not have been ideal to investigate associations with these disease outcomes.

To further compare our mLOY findings with those previously reported, we restricted our analyses to male participants over the age of 65 years old but did not observe evidence for associations of mLOY with Alzheimer’s disease, major cardiovascular events or age-related macular degeneration (Additional file 1: Table S9). Our detection method also included phase-based data to increase sensitivity to detect lower frequency mLOY clones, which may have reduced the effect size of associations with disease risk. To further explore the impact of clonal frequency on disease risk, we performed sensitivity analyses using different cell fraction thresholds (0.03, 0.1, and 0.2), but did not observe evidence for mLOY associations with incidence of closely matching disease codes of the previously reported diseases above passing phenome-wide significance level (Additional file 1: Table S10). Finally, our modeling approach investigated incident disease risk in which some previously reported mLOY associations were for prevalent disease and adjusted for an expanded list of confounders in an attempt to as comprehensively as possible rule out potential biases. For example, we derived and adjusted for more granular smoking covariates (25 levels, Additional file 1: Table S11) as well as modeled age with both age and age^2^ to account for potential non-linear relationships with age (Additional file 1: Tables S12).

Interestingly, we observed negative associations between mLOY and several metabolic-related diseases. We performed a series of exploratory analyses to investigate these protective associations further and found that adjustment for BMI substantially attenuated many of these associations; however, risk for primary hypertension and retinal diseases associated with metabolic syndrome remained statistically significant. MR analyses using previously reported mLOY variants discovered by GWAS [46] added additional supporting evidence to confirm the associations with primary hypertension and retinal diseases. While it is reassuring that the association between retinal diseases and mLOY were confirmed in an MR analysis, we note that the mLOY discovery GWAS and this PheWAS contain sample overlap which could result in bias of the MR results toward the observed PheWAS result. Biologic mechanisms linking mLOY with hypertension and retinal diseases are unclear and future studies in independent populations are needed to confirm these observed relationships.

In our PheWAS, we compared three models with respect to smoking adjustment: (1) no smoking adjustment, (2) adjusting for 3 smoking categories (never, former, and current), and (3) with a 25-level detailed smoking adjustment. The results in Additional file 1: Table S11 demonstrates that some observed associations between mLOY and diseases using models that did not carefully adjust for smoking behavior are due to residual confounding, for example some behavioral outcomes were completely confounded by smoking. Similarly, we also ran models further examining the impact of age adjustment. We ran the following models: (1) unadjusted for age, (2) only adjusting for linear age, and (3) adjusting for both a linear and squared age term. Results with no adjustment for age demonstrated a host of spurious associations due to confounding by age (Additional file 1: Table S12). While linear age adjustment removed several of these spurious findings, a few associations such as mental and behavioral disorders due to opioids, arthrosis, and polyuria remained nominally significant indicating evidence of residual confounding, perhaps due to age. Our results suggest that including both a linear and squared term for age adjustment resulted in robust statistical adjustment for potential confounding due to age.

We conducted exploratory analyses that included prevalent disease. While we identified several potential associations between prevalent disease and mCAs, the incident disease associations did not always support these findings. In addition, as the timing of prevalent disease diagnosis is unknown relative to the onset of mosaicism, it is not possible to time the relationship between disease and detectible mCA in the UK Biobank (i.e., the order could not be conclusively defined for diseases). We included preliminary results from a PheWAS of prevalent conditions as exploratory analyses for hypothesis generation and initial evidence for designing future investigations. We highlighted similarities across autosomal and sex chromosome mosaicism, but recommend caution be taken in the interpretation of these findings and stress the critical need for further replication in independent datasets.

Likewise, we performed PheWAS on medication use, but it is difficult to separate the relationship between medications and diagnoses in these results. For example, someone with a diabetes diagnosis is likely to be taking a diabetes medication such as metformin. While the mechanism of metformin is still being studied, it is possible that metformin-induced inhibition of gluconeogenesis and the mTOR pathway [35, 38] could have impacts on mCA clonal expansion [10, 47].

There are limitations of our study that should be taken into account when interpreting the associations reported herein between mCAs and diseases. First, although the date of diagnosis provides a certain level of temporality between mosaic events and diseases, we are unaware of the time period when initial symptoms and onset of diseases occurred. The onset of disease might predate the onset of mosaicism even though the diagnoses occurred much later. Second, we chose the first occurrence of each disease as a defining marker of onset. For diseases or treatments that are ongoing or chronically reoccur, factoring in multiple episodes of that particular disease might further increase the power to detect associations. Third, there may be some inaccuracies or underreporting in the first occurrence data in the UK Biobank due to (1) the primary care data is still an interim release with ~ 45% of the participants available, (2) the completeness of inpatient data varies by geographical location with England dating back to 1997, Wales to 1998, and Scotland to 1981, and (3) self-report information from participants are susceptible to inaccuracies and biases. Finally, conditions which do not result in primary care or inpatient admissions are inevitably missing and not captured in the analyses.

## Conclusion

In the current investigation, we report evidence for a broad spectrum of associations between mCAs and first occurrence of diseases that varies by type of mCA and highlight the critical importance of careful covariate adjustment to minimize confounding. Our findings suggest mCAs could be linked to a spectrum of health outcomes and as such future more focused studies are needed to identify important etiologic relationships with potential clinical utility for assessment of disease risk.

## Supplementary Information

**Additional file 1.** Additional figures and tables.

## Data Availability

The data reported in this paper are available by application directly to the UK Biobank. Statistically significant associations between mosaicism and health outcomes as well as medication are provided in the manuscript. Software code in R for the analyses is available upon request.
