# Supplementary material for: Incident disease associations with mosaic chromosomal alterations on autosomes, X and Y chromosomes: insights from a phenome-wide association study in the UK Biobank
Source: Cell Biosci. 2021 Jul 23;11:143. doi: 10.1186/s13578-021-00651-z (PMC8299574; doi:10.1186/s13578-021-00651-z)
Supplement: Supplementary file 1 — Additional file 1. Additional figures and tables. [file 13578_2021_651_MOESM1_ESM.docx]

**Additional File**

**Figure S1.** Circos plot of previously reported autosomal mCAs (Loh *et al*, 2018, 2020). The outer to inner tracks represent mCAs categories: copy number neutral, copy number loss, copy number gain, and undetermined copy number. Each bar represents 5Mb regions, and the height of bars represents the relative frequency of overlapping mosaic copy number variation within each of the four categories (copy number neutral loss of heterozygosity (LOH), loss, gain, and undetermined).


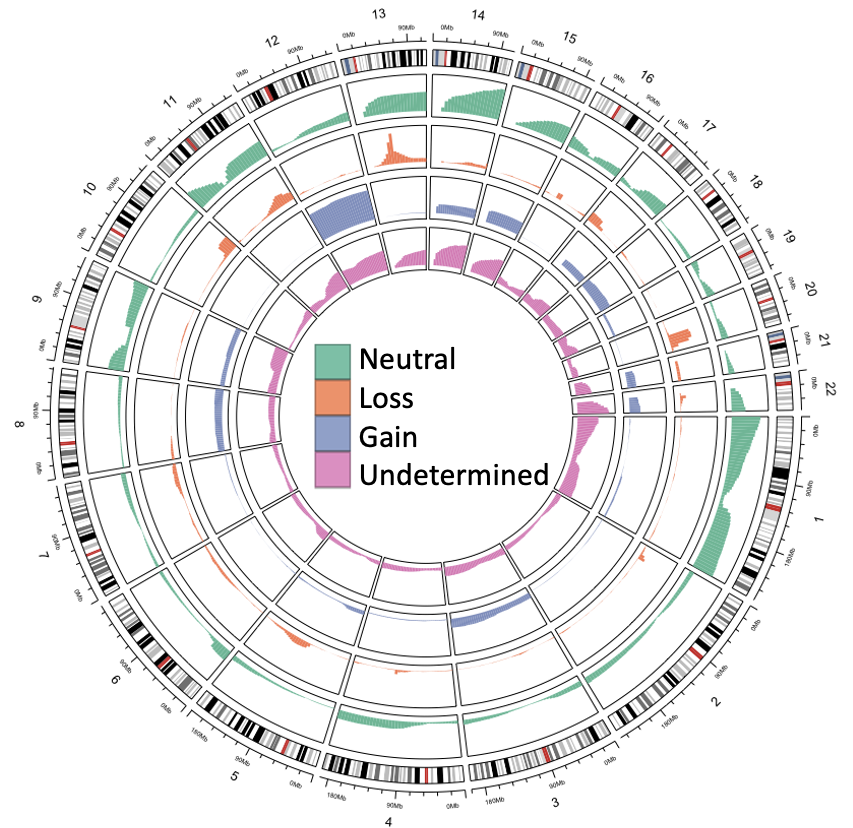


**Figure S2.** Manhattan plots of prevalent disease diagnoses and mCAs in the UK Biobank adjusting for age, age^2^, detailed 25-level smoking, and sex (autosomal mCA analysis only). The red line represents the Bonferroni-corrected, PheWAS significance level. The dashed, blue line represents the suggestive threshold (1/number of diseases tested). A maximum of the ten most significant medication codes above the suggestive threshold are labeled. PheWAS results are for:

**(a)** Autosomal mCAs: 1,281 diseases (PheWAS significance level: 3.90 x 10^-5^ , suggestive line: 7.81 x 10^-4^ ) ; C85 Other and unspecified types of non-Hodgkin’s lymphoma, C91 Lymphoid leukaemia, D45 Polycythaemia vera, D46 Myelodysplastic syndromes, D47 Other neoplasms of uncertain or unknown behaviour of lymphoid, haematopoietic and related tissue, E78 Disorders of lipoprotein metabolism and other lipidaemias, I10 Essential (primary) hypertension, M19 Other arthrosis, N40 Hyperplasia of prostate, Z37 Outcome of delivery.

**(b)** mLOX: 1,112 diseases (PheWAS significance level: 4.50 x 10^-5^ , suggestive line:8.99 x 10^-4^ ); C44 Other malignant neoplasms of skin, E78 Disorders of lipoprotein metabolism and other lipidaemias, H26 Other cataract, I10 Essential (primary) hypertension, I20 Angina pectoris, K44 Diaphragmatic hernia, M19 Other arthrosis, N95 Menopausal and other perimenopausal disorders, Z30 Contraceptive management, and Z37 Outcome of delivery.

and

**(c)** mLOY: 1,097 diseases (PheWAS significance level: 4.56 x 10^-5^ , suggestive line:9.11 x 10^-4^ ); E78 Disorders of lipoprotein metabolism and other lipidaemias, F17 Mental and behavioural disorders due to the use of tobacco, I10 Essential (primary) hypertension, I20 Angina pectoris, I21 Acute myocardial infarction, I25 Chronic ischaemic heart disease, M19 Other arthrosis, N40 Hyperplasia of prostate, Z30 Contraceptive management, and Z72 Problems related to lifestyle.


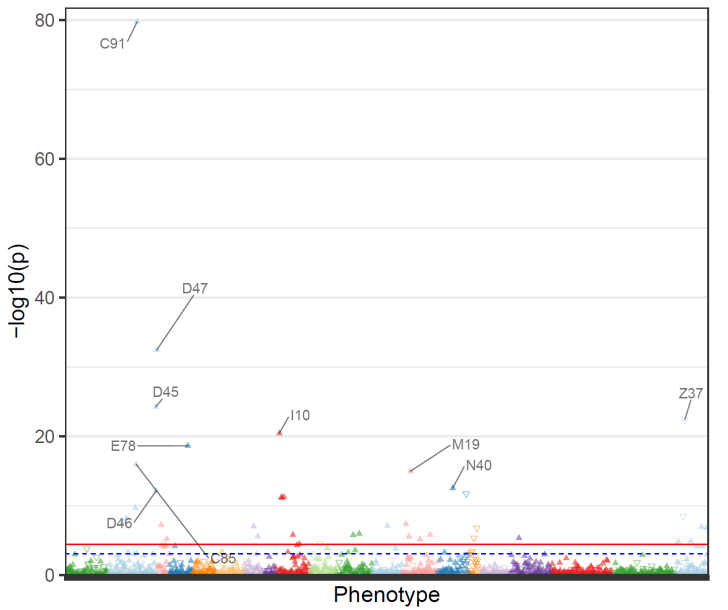


**(b)**


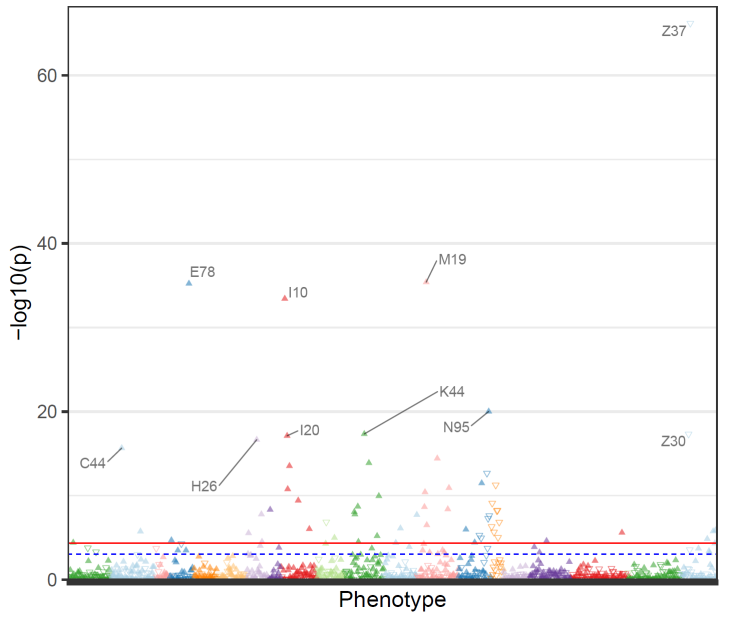


**c)**


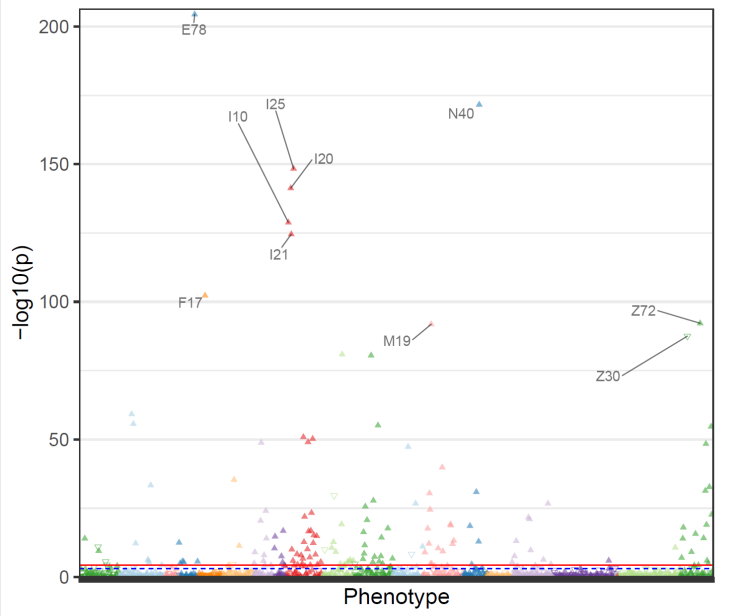


**Table S1.** Effect estimates for non-cancer diseases and conditions with autosomal mCA associations stratified by prior cancer history. All analyses are adjusted for age, age^2^, detailed 25-level smoking, and sex.

|  | Prior history of cancer | |  | No prior cancer history | |
| --- | --- | --- | --- | --- | --- |
| Disease | Odds Ratio [95% Confidence Interval] | P-value |  | Odds Ratio [95% Confidence Interval] | P-value |
| D70 agranulocytosis | 1.82 [1.41, 2.30] | 1.55×10^-6^ |  | 2.16 [1.44, 3.39] | 4.23×10^-4^ |
| D69 purpura and other haemorrhagic conditions | 1.58 [1.27, 1.93] | 1.78×10^-5^ |  | 0.91 [0.53, 1.67] | 0.747 |
| A41 other septicaemia | 1.14 [0.99, 1.31] | 0.069 |  | 1.61 [0.96, 2.91] | 0.09 |
| D80 immunodeficiency with predominantly antibody defects | 1.69 [0.71, 3.37] | 0.182 |  | 1.26 [0.18, 14.07] | 0.826 |
| D75 other diseases of blood and blood-forming organs | 1.94 [1.43, 2.58] | 8.62×10^-6^ |  | 1.72 [0.86, 3.76] | 0.149 |
| D72 other disorders of white blood cells | 2.18 [1.55, 2.98] | 2.60×10^-6^ |  | 1.21 [0.46, 3.92] | 0.722 |
| J18 pneumonia, organism unspecified | 1.07 [0.97, 1.18] | 0.171 |  | 1.45 [1.01, 2.16] | 0.055 |
| D61 other aplastic anaemias | 1.81 [1.19, 2.63] | 0.003 |  | 1.29 [0.47, 4.56] | 0.657 |
| J90 pleural effusion, not elsewhere classified | 1.06 [0.93, 1.21] | 0.368 |  | 1.56 [0.93, 2.82] | 0.117 |
| J98 other respiratory disorders | 1.13 [1.01, 1.26] | 0.026 |  | 1.42 [0.92, 2.34] | 0.138 |
| B25 cytomegaloviral disease | 1.45 [0.35, 3.93] | 0.533 |  | NA* | 0.998 |
| N17 acute renal failure | 1.01 [0.91, 1.13] | 0.838 |  | 4.90 [1.55, 29.77] | 0.026 |
| E87 other disorders of fluid, electrolyte and acid-base balance | 1.01 [0.91, 1.12] | 0.829 |  | 1.28 [0.80, 2.19] | 0.341 |
| D64 other anaemias | 1.05 [0.95, 1.16] | 0.323 |  | 1.16 [0.88, 1.55] | 0.316 |
| D59 acquired haemolytic anaemia | 1.85 [0.72, 3.92] | 0.147 |  | 0.28 [0.07, 1.05] | 0.057 |
| B96 other bacterial agents as the cause of diseases classified to other chapters | 0.96 [0.84, 1.10] | 0.576 |  | 1.33 [0.95, 1.91] | 0.112 |
| E83 disorders of mineral metabolism | 1.04 [0.85, 1.25] | 0.687 |  | 0.84 [0.46, 1.68] | 0.581 |
| G72 other myopathies | 2.79 [1.62, 4.48] | 6.98×10^-5^ |  | 1.59 [0.16, 57.25] | 0.736 |

* : The odds ratio could not be estimated because all mLOY individuals in this group had cytomegaloviral infection

**Table S2.** Statistically significant associations for autosomal mCAs and incident disease associations. Only PheWAS significant associations are reported (P<3.88x10^-5^ for autosomal, P<4.43x10^-5^ for mLOX, and P<4.39x10^-5^ for mLOY). All PheWAS associations are adjusted for age, age^2^, smoking, genetic ancestry, and sex (only for autosomal mCA analyses).

| **mCA** | **Disease** | **Odds Ratio**  **[95% Confidence Interval]** | **P-value** |
| --- | --- | --- | --- |
| Autosome | C91 Lymphoid leukaemia | 23.76 [20.23-27.91] | <5×10-324 |
| Autosome | D47 Other neoplasms of uncertain or unknown behaviour of lymphoid, haematopoietic and related tissue | 5.34 [4.43-6.45] | 4.15E-68 |
| Autosome | C83 Diffuse non-Hodgkin's lymphoma | 4.62 [3.88-5.5] | 1.09E-65 |
| Autosome | D45 Polycythaemia vera | 12.24 [9.09-16.48] | 4.32E-61 |
| Autosome | C85 Other and unspecified types of non-Hodgkin's lymphoma | 4.28 [3.55-5.14] | 1.49E-53 |
| Autosome | D70 Agranulocytosis | 2.25 [2.02-2.5] | 6.25E-51 |
| Autosome | D46 Myelodysplastic syndromes | 6.19 [4.72-8.13] | 1.69E-39 |
| Autosome | C92 Myeloid leukaemia | 5.18 [3.99-6.71] | 2.13E-35 |
| Autosome | R16 Hepatomegaly and splenomegaly, not elsewhere classified | 3.79 [3-4.78] | 7.55E-29 |
| Autosome | D69 Purpura and other haemorrhagic conditions | 2.02 [1.77-2.3] | 1.08E-25 |
| Autosome | A41 Other septicaemia | 1.55 [1.42-1.68] | 1.48E-25 |
| Autosome | D75 Other diseases of blood and blood-forming organs | 2.77 [2.27-3.38] | 8.38E-24 |
| Autosome | D80 Immunodeficiency with predominantly antibody defects | 5.41 [3.89-7.52] | 9.80E-24 |
| Autosome | C95 Leukaemia of unspecified cell type | 13.35 [7.42-24.02] | 5.24E-18 |
| Autosome | D72 Other disorders of white blood cells | 2.68 [2.12-3.38] | 1.94E-16 |
| Autosome | J18 Pneumonia, organism unspecified | 1.32 [1.24-1.41] | 2.25E-16 |
| Autosome | C94 Other leukaemias of specified cell type | 15.14 [7.29-31.42] | 3.01E-13 |
| Autosome | D61 Other aplastic anaemias | 2.25 [1.78-2.83] | 5.96E-12 |
| Autosome | Z51 Other medical care | 1.24 [1.16-1.32] | 1.32E-11 |
| Autosome | C84 Peripheral and cutaneous T-cell lymphomas | 4.34 [2.82-6.68] | 2.76E-11 |
| Autosome | J90 Pleural effusion, not elsewhere classified | 1.29 [1.18-1.41] | 8.28E-09 |
| Autosome | C88 Malignant immunoproliferative diseases | 4.17 [2.54-6.83] | 1.46E-08 |
| Autosome | C82 Follicular [nodular] non-Hodgkin's lymphoma | 2.69 [1.9-3.81] | 2.25E-08 |
| Autosome | Z85 Personal history of malignant neoplasm | 1.2 [1.13-1.29] | 2.98E-08 |
| Autosome | J98 Other respiratory disorders | 1.26 [1.16-1.37] | 3.23E-08 |
| Autosome | B25 Cytomegaloviral disease | 3.71 [2.32-5.94] | 4.75E-08 |
| Autosome | Y43 Primarily systemic agents | 1.58 [1.34-1.86] | 5.37E-08 |
| Autosome | N17 Acute renal failure | 1.23 [1.14-1.32] | 6.75E-08 |
| Autosome | E87 Other disorders of fluid, electrolyte and acid-base balance | 1.22 [1.13-1.32] | 1.17E-07 |
| Autosome | D64 Other anaemias | 1.21 [1.12-1.3] | 3.07E-07 |
| Autosome | Z94 Transplanted organ and tissue status | 2 [1.51-2.66] | 1.67E-06 |
| Autosome | B96 Other bacterial agents as the cause of diseases classified to other chapters | 1.24 [1.13-1.36] | 4.68E-06 |
| Autosome | R50 Fever of unknown origin | 1.41 [1.22-1.64] | 5.12E-06 |
| Autosome | D59 Acquired haemolytic anaemia | 2.97 [1.86-4.74] | 5.45E-06 |
| Autosome | R60 Oedema, not elsewhere classified | 1.49 [1.25-1.78] | 1.01E-05 |
| Autosome | E83 Disorders of mineral metabolism | 1.3 [1.15-1.47] | 1.87E-05 |
| Autosome | R22 Localised swelling, mass and lump of skin and subcutaneous tissue | 1.67 [1.32-2.12] | 2.50E-05 |
| Autosome | T86 Failure and rejection of transplanted organs and tissues | 2.61 [1.66-4.1] | 3.11E-05 |
| Autosome | G72 Other myopathies | 2.46 [1.61-3.75] | 3.12E-05 |

**Table S3.** Statistically significant associations between mCAs on each autosome and incident disease risk adjusted for age, age^2^, smoking, and sex.

| mCAs | Disease | Odds Ratio [95% Confidence Interval] | P-value |
| --- | --- | --- | --- |
| chr1 | C83 Diffuse non-Hodgkin's lymphoma | 8.07 [5.61-11.59] | 1.67×10^-29^ |
| chr1 | C85 Other and unspecified types of non-Hodgkin's lymphoma | 6.23 [4.1-9.46] | 9.91×10^-18^ |
| chr1 | C91 Lymphoid leukaemia | 6.31 [3.88-10.26] | 1.05×10^-13^ |
| chr1 | D70 Agranulocytosis | 2.68 [2.03-3.55] | 5.44×10^-12^ |
| chr1 | D47 Other neoplasms of uncertain or unknown behaviour of lymphoid, haematopoietic and related tissue | 4.71 [2.81-7.87] | 3.62×10^-9^ |
| chr1 | R16 Hepatomegaly and splenomegaly, not elsewhere classified | 5.06 [2.91-8.79] | 8.97×10^-9^ |
| chr1 | C82 Follicular [nodular] non-Hodgkin's lymphoma | 6.31 [3.25-12.26] | 5.58×10^-8^ |
| chr1 | C84 Peripheral and cutaneous T-cell lymphomas | 9.05 [3.99-20.55] | 1.37×10^-7^ |
| chr1 | D80 Immunodeficiency with predominantly antibody defects | 7 [3.29-14.88] | 4.31×10^-7^ |
| chr1 | Y43 Primarily systemic agents | 2.56 [1.75-3.76] | 1.44×10^-6^ |
| chr1 | Z51 Other medical care | 1.49 [1.26-1.77] | 4.04×10^-6^ |
| chr1 | C92 Myeloid leukaemia | 4.75 [2.35-9.6] | 1.41×10^-5^ |
| chr2 | C91 Lymphoid leukaemia | 15.24 [8.89-26.12] | 3.76×10^-23^ |
| chr2 | R04 Haemorrhage from respiratory passages | 3.12 [1.86-5.23] | 1.54×10^-5^ |
| chr3 | C83 Diffuse non-Hodgkin's lymphoma | 21.99 [15.18-31.84] | 3.65×10^-60^ |
| chr3 | C85 Other and unspecified types of non-Hodgkin's lymphoma | 16.18 [10.49-24.95] | 2.36×10^-36^ |
| chr3 | C88 Malignant immunoproliferative diseases | 42.88 [20.72-88.74] | 4.11×10^-24^ |
| chr3 | C91 Lymphoid leukaemia | 12.82 [7.34-22.39] | 3.16×10^-19^ |
| chr3 | D70 Agranulocytosis | 4.39 [3.02-6.38] | 1.06×10^-14^ |
| chr3 | Z94 Transplanted organ and tissue status | 9.89 [5.26-18.58] | 1.08×10^-12^ |
| chr3 | D82 Immunodeficiency associated with other major defects | 63.88 [19.16-213] | 1.33×10^-11^ |
| chr3 | C82 Follicular [nodular] non-Hodgkin's lymphoma | 13.35 [6.27-28.39] | 1.71×10^-11^ |
| chr3 | D80 Immunodeficiency with predominantly antibody defects | 14.12 [5.79-34.45] | 5.90×10^-9^ |
| chr3 | R50 Fever of unknown origin | 3.74 [2.36-5.92] | 1.72×10^-8^ |
| chr3 | A41 Other septicaemia | 2.49 [1.8-3.44] | 3.48×10^-8^ |
| chr3 | N27 Small kidney of unknown cause | 26.19 [8.19-83.78] | 3.71×10^-8^ |
| chr3 | D61 Other aplastic anaemias | 6.2 [3.19-12.05] | 7.15×10^-8^ |
| chr3 | D47 Other neoplasms of uncertain or unknown behaviour of lymphoid, haematopoietic and related tissue | 6.79 [3.36-13.72] | 9.16×10^-8^ |
| chr3 | R16 Hepatomegaly and splenomegaly, not elsewhere classified | 7.61 [3.59-16.14] | 1.19×10^-7^ |
| chr3 | X12 Contact with other hot fluids | 34.34 [8.22-143.39] | 1.24×10^-6^ |
| chr3 | Y43 Primarily systemic agents | 3.53 [2.03-6.13] | 7.58×10^-6^ |
| chr3 | D58 Other hereditary haemolytic anaemias | 12.98 [4.12-40.92] | 1.21×10^-5^ |
| chr3 | E83 Disorders of mineral metabolism | 2.61 [1.69-4.05] | 1.73×10^-5^ |
| chr3 | Z51 Other medical care | 1.78 [1.36-2.33] | 2.39×10^-5^ |
| chr4 | D70 Agranulocytosis | 5.74 [3.98-8.27] | 7.71×10^-21^ |
| chr4 | D46 Myelodysplastic syndromes | 21.16 [10.77-41.55] | 7.84×10^-19^ |
| chr4 | C91 Lymphoid leukaemia | 13.38 [7.3-24.53] | 5.07×10^-17^ |
| chr4 | D69 Purpura and other haemorrhagic conditions | 5.84 [3.83-8.91] | 2.63×10^-16^ |
| chr4 | C92 Myeloid leukaemia | 15.6 [7.67-31.72] | 3.37×10^-14^ |
| chr4 | D72 Other disorders of white blood cells | 9.57 [4.92-18.61] | 2.85×10^-11^ |
| chr4 | A41 Other septicaemia | 2.99 [2.15-4.17] | 8.53×10^-11^ |
| chr4 | C93 Monocytic leukaemia | 37.98 [11.65-123.74] | 1.59×10^-9^ |
| chr4 | C85 Other and unspecified types of non-Hodgkin's lymphoma | 7.69 [3.95-14.95] | 1.85×10^-9^ |
| chr4 | C84 Peripheral and cutaneous T-cell lymphomas | 19.73 [7.24-53.77] | 5.53×10^-9^ |
| chr4 | I74 Arterial embolism and thrombosis | 5.81 [2.97-11.39] | 2.89×10^-7^ |
| chr4 | C75 Malignant neoplasm of other endocrine glands and related structures | 40.77 [9.72-171.05] | 4.02×10^-7^ |
| chr4 | C86 Other specified types of T/NK-cell lymphoma | 41.86 [9.83-178.19] | 4.36×10^-7^ |
| chr4 | Z51 Other medical care | 1.98 [1.49-2.62] | 2.22×10^-6^ |
| chr4 | R50 Fever of unknown origin | 3.39 [1.99-5.77] | 7.32×10^-6^ |
| chr4 | D65 Disseminated intravascular coagulation [defibrination syndrome] | 23.86 [5.78-98.56] | 1.17×10^-5^ |
| chr4 | C82 Follicular [nodular] non-Hodgkin's lymphoma | 9.02 [3.35-24.32] | 1.39×10^-5^ |
| chr4 | J90 Pleural effusion, not elsewhere classified | 2.26 [1.56-3.26] | 1.43×10^-5^ |
| chr4 | J18 Pneumonia, organism unspecified | 1.97 [1.45-2.69] | 1.79×10^-5^ |
| chr4 | L53 Other erythematous conditions | 5.55 [2.47-12.47] | 3.31×10^-5^ |
| chr5 | D46 Myelodysplastic syndromes | 34.16 [18.92-61.7] | 1.17×10^-31^ |
| chr5 | C92 Myeloid leukaemia | 23.23 [12.27-43.97] | 4.52×10^-22^ |
| chr5 | Z94 Transplanted organ and tissue status | 13.64 [7.25-25.65] | 5.26×10^-16^ |
| chr5 | T86 Failure and rejection of transplanted organs and tissues | 25.96 [11.46-58.82] | 6.01×10^-15^ |
| chr5 | D70 Agranulocytosis | 4.46 [2.9-6.85] | 9.41×10^-12^ |
| chr5 | C91 Lymphoid leukaemia | 11.19 [5.52-22.67] | 2.02×10^-11^ |
| chr5 | R50 Fever of unknown origin | 4.89 [3.05-7.85] | 4.59×10^-11^ |
| chr5 | D61 Other aplastic anaemias | 8.96 [4.6-17.43] | 1.09×10^-10^ |
| chr5 | A41 Other septicaemia | 3.13 [2.21-4.44] | 1.45×10^-10^ |
| chr5 | C85 Other and unspecified types of non-Hodgkin's lymphoma | 8.18 [4.05-16.53] | 4.86×10^-9^ |
| chr5 | D69 Purpura and other haemorrhagic conditions | 4.28 [2.55-7.18] | 3.74×10^-8^ |
| chr5 | N15 Other renal tubulo-interstitial diseases | 25.09 [7.91-79.6] | 4.47×10^-8^ |
| chr5 | C83 Diffuse non-Hodgkin's lymphoma | 6.75 [3.19-14.31] | 6.25×10^-7^ |
| chr5 | B25 Cytomegaloviral disease | 18.04 [5.73-56.79] | 7.70×10^-7^ |
| chr6 | C91 Lymphoid leukaemia | 17.56 [10.59-29.12] | 1.24×10^-28^ |
| chr6 | C83 Diffuse non-Hodgkin's lymphoma | 12.56 [7.7-20.48] | 3.64×10^-24^ |
| chr6 | C85 Other and unspecified types of non-Hodgkin's lymphoma | 11.72 [6.98-19.7] | 1.40×10^-20^ |
| chr6 | D70 Agranulocytosis | 4.13 [2.81-6.09] | 7.15×10^-13^ |
| chr6 | C82 Follicular [nodular] non-Hodgkin's lymphoma | 13.73 [6.46-29.19] | 9.87×10^-12^ |
| chr6 | D82 Immunodeficiency associated with other major defects | 40.25 [9.49-170.78] | 5.41×10^-7^ |
| chr6 | A41 Other septicaemia | 2.36 [1.68-3.32] | 7.46×10^-7^ |
| chr6 | D80 Immunodeficiency with predominantly antibody defects | 11.73 [4.35-31.65] | 1.17×10^-6^ |
| chr6 | D47 Other neoplasms of uncertain or unknown behaviour of lymphoid, haematopoietic and related tissue | 6.22 [2.94-13.18] | 1.77×10^-6^ |
| chr6 | C88 Malignant immunoproliferative diseases | 16.42 [5.19-51.93] | 1.91×10^-6^ |
| chr6 | Z92 Personal history of medical treatment | 1.71 [1.35-2.17] | 7.83×10^-6^ |
| chr6 | B25 Cytomegaloviral disease | 13.59 [4.32-42.74] | 8.10×10^-6^ |
| chr6 | G53 Cranial nerve disorders in diseases classified elsewhere | 13.4 [4.25-42.24] | 9.44×10^-6^ |
| chr6 | R61 Hyperhidrosis | 6.73 [2.77-16.3] | 2.45×10^-5^ |
| chr7 | C92 Myeloid leukaemia | 22.94 [12.12-43.43] | 6.53×10^-22^ |
| chr7 | D46 Myelodysplastic syndromes | 22.06 [10.81-45.02] | 1.91×10^-17^ |
| chr7 | C93 Monocytic leukaemia | 66.35 [23.55-186.88] | 2.03×10^-15^ |
| chr7 | C83 Diffuse non-Hodgkin's lymphoma | 10.76 [5.88-19.69] | 1.26×10^-14^ |
| chr7 | C91 Lymphoid leukaemia | 12.59 [6.46-24.54] | 1.01×10^-13^ |
| chr7 | C85 Other and unspecified types of non-Hodgkin's lymphoma | 10.28 [5.46-19.34] | 5.11×10^-13^ |
| chr7 | D70 Agranulocytosis | 4.71 [3.09-7.18] | 5.50×10^-13^ |
| chr7 | C95 Leukaemia of unspecified cell type | 53.3 [16.43-172.89] | 3.54×10^-11^ |
| chr7 | Z94 Transplanted organ and tissue status | 10.68 [5.28-21.6] | 4.28×10^-11^ |
| chr7 | D61 Other aplastic anaemias | 6.89 [3.25-14.61] | 4.90×10^-7^ |
| chr7 | B25 Cytomegaloviral disease | 17.91 [5.69-56.42] | 8.28×10^-7^ |
| chr7 | R16 Hepatomegaly and splenomegaly, not elsewhere classified | 7.23 [2.98-17.55] | 1.23×10^-5^ |
| chr7 | T86 Failure and rejection of transplanted organs and tissues | 12.54 [3.99-39.37] | 1.49×10^-5^ |
| chr8 | C85 Other and unspecified types of non-Hodgkin's lymphoma | 21.23 [13.46-33.49] | 2.06×10^-39^ |
| chr8 | C83 Diffuse non-Hodgkin's lymphoma | 18.94 [11.88-30.21] | 4.86×10^-35^ |
| chr8 | D70 Agranulocytosis | 6.26 [4.31-9.09] | 4.60×10^-22^ |
| chr8 | C84 Peripheral and cutaneous T-cell lymphomas | 42.02 [19.49-90.6] | 1.46×10^-21^ |
| chr8 | A41 Other septicaemia | 3.85 [2.79-5.32] | 2.10×10^-16^ |
| chr8 | C88 Malignant immunoproliferative diseases | 38.23 [15.48-94.41] | 2.77×10^-15^ |
| chr8 | D46 Myelodysplastic syndromes | 19.75 [9.24-42.23] | 1.41×10^-14^ |
| chr8 | B25 Cytomegaloviral disease | 30.78 [12.55-75.49] | 7.05×10^-14^ |
| chr8 | C91 Lymphoid leukaemia | 12.53 [6.43-24.42] | 1.12×10^-13^ |
| chr8 | D61 Other aplastic anaemias | 10.16 [5.39-19.13] | 7.01×10^-13^ |
| chr8 | R16 Hepatomegaly and splenomegaly, not elsewhere classified | 12.55 [6.2-25.43] | 2.14×10^-12^ |
| chr8 | C82 Follicular [nodular] non-Hodgkin's lymphoma | 15.84 [7.02-35.77] | 2.93×10^-11^ |
| chr8 | Y43 Primarily systemic agents | 5.51 [3.28-9.24] | 1.01×10^-10^ |
| chr8 | D69 Purpura and other haemorrhagic conditions | 4.69 [2.84-7.75] | 1.56×10^-9^ |
| chr8 | Z51 Other medical care | 2.37 [1.79-3.13] | 1.65×10^-9^ |
| chr8 | Z94 Transplanted organ and tissue status | 9.6 [4.53-20.36] | 3.58×10^-9^ |
| chr8 | D47 Other neoplasms of uncertain or unknown behaviour of lymphoid, haematopoietic and related tissue | 8.37 [3.95-17.75] | 3.05×10^-8^ |
| chr8 | C95 Leukaemia of unspecified cell type | 36.64 [8.83-152.01] | 7.00×10^-7^ |
| chr8 | J90 Pleural effusion, not elsewhere classified | 2.53 [1.73-3.7] | 1.75×10^-6^ |
| chr8 | R58 Haemorrhage, not elsewhere classified | 23.48 [5.72-96.35] | 1.18×10^-5^ |
| chr8 | B96 Other bacterial agents as the cause of diseases classified to other chapters | 2.35 [1.58-3.5] | 2.65×10^-5^ |
| chr8 | C69 Malignant neoplasm of eye and adnexa | 20.08 [4.92-81.97] | 2.93×10^-5^ |
| chr8 | J17 Pneumonia in diseases classified elsewhere | 11.49 [3.65-36.14] | 2.98×10^-5^ |
| chr9 | D45 Polycythaemia vera | 182.61 [133.26-250.23] | 3.05×10^-230^ |
| chr9 | D47 Other neoplasms of uncertain or unknown behaviour of lymphoid, haematopoietic and related tissue | 33.92 [25.85-44.52] | 1.77×10^-142^ |
| chr9 | D75 Other diseases of blood and blood-forming organs | 24.86 [19.07-32.4] | 7.32×10^-125^ |
| chr9 | R16 Hepatomegaly and splenomegaly, not elsewhere classified | 11.39 [7.09-18.32] | 1.03×10^-23^ |
| chr9 | D69 Purpura and other haemorrhagic conditions | 4.93 [3.61-6.74] | 1.19×10^-23^ |
| chr9 | C94 Other leukaemias of specified cell type | 69.02 [26.1-182.5] | 1.39×10^-17^ |
| chr9 | C92 Myeloid leukaemia | 9.01 [4.78-16.97] | 1.01×10^-11^ |
| chr9 | D46 Myelodysplastic syndromes | 9.66 [4.95-18.85] | 2.98×10^-11^ |
| chr9 | D73 Diseases of spleen | 6.44 [3.7-11.19] | 4.06×10^-11^ |
| chr9 | D70 Agranulocytosis | 2.74 [1.93-3.88] | 1.43×10^-8^ |
| chr9 | D58 Other hereditary haemolytic anaemias | 12.52 [5.11-30.67] | 3.19×10^-8^ |
| chr9 | T13 Other injuries of lower limb, level unspecified | 20.84 [6.48-66.96] | 3.43×10^-7^ |
| chr9 | C85 Other and unspecified types of non-Hodgkin's lymphoma | 4.47 [2.46-8.14] | 9.56×10^-7^ |
| chr9 | J18 Pneumonia, organism unspecified | 1.73 [1.39-2.17] | 1.25×10^-6^ |
| chr9 | C91 Lymphoid leukaemia | 4.96 [2.56-9.62] | 2.15×10^-6^ |
| chr9 | D61 Other aplastic anaemias | 4.24 [2.33-7.73] | 2.28×10^-6^ |
| chr9 | I85 Oesophageal varices | 4.46 [2.3-8.64] | 9.53×10^-6^ |
| chr9 | C83 Diffuse non-Hodgkin's lymphoma | 3.78 [2.02-7.08] | 3.25×10^-5^ |
| chr10 | C83 Diffuse non-Hodgkin's lymphoma | 9.69 [5.15-18.22] | 1.83×10^-12^ |
| chr10 | C85 Other and unspecified types of non-Hodgkin's lymphoma | 7.92 [3.92-16] | 8.10×10^-9^ |
| chr10 | C91 Lymphoid leukaemia | 8.71 [3.87-19.59] | 1.71×10^-7^ |
| chr10 | D70 Agranulocytosis | 3.09 [1.91-5.03] | 4.93×10^-6^ |
| chr10 | E13 Other specified diabetes mellitus | 10 [3.71-26.96] | 5.29×10^-6^ |
| chr10 | C82 Follicular [nodular] non-Hodgkin's lymphoma | 9.56 [3.55-25.74] | 7.99×10^-6^ |
| chr10 | T86 Failure and rejection of transplanted organs and tissues | 11.7 [3.73-36.77] | 2.53×10^-5^ |
| chr11 | C91 Lymphoid leukaemia | 13.58 [9.41-19.61] | 4.21×10^-44^ |
| chr11 | C34 Malignant neoplasm of bronchus and lung | 2.33 [1.6-3.41] | 1.26×10^-5^ |
| chr12 | C91 Lymphoid leukaemia | 41.01 [31.5-53.38] | 9.24×10^-168^ |
| chr12 | C85 Other and unspecified types of non-Hodgkin's lymphoma | 15.1 [10.83-21.06] | 1.32×10^-57^ |
| chr12 | C83 Diffuse non-Hodgkin's lymphoma | 14.46 [10.41-20.07] | 3.09×10^-57^ |
| chr12 | D70 Agranulocytosis | 4.11 [3.1-5.43] | 5.36×10^-23^ |
| chr12 | D80 Immunodeficiency with predominantly antibody defects | 17.04 [9.25-31.38] | 8.89×10^-20^ |
| chr12 | R16 Hepatomegaly and splenomegaly, not elsewhere classified | 8.08 [4.73-13.79] | 2.01×10^-14^ |
| chr12 | A41 Other septicaemia | 2.45 [1.93-3.1] | 1.34×10^-13^ |
| chr12 | C38 Malignant neoplasm of heart, mediastinum and pleura | 26.62 [10.58-66.98] | 3.14×10^-12^ |
| chr12 | D84 Other immunodeficiencies | 17.86 [6.51-49] | 2.18×10^-8^ |
| chr12 | Z51 Other medical care | 1.73 [1.42-2.1] | 5.10×10^-8^ |
| chr12 | C92 Myeloid leukaemia | 6.72 [3.32-13.59] | 1.18×10^-7^ |
| chr12 | R59 Enlarged lymph nodes | 3.24 [2.08-5.07] | 2.26×10^-7^ |
| chr12 | D46 Myelodysplastic syndromes | 7.04 [3.31-14.96] | 3.96×10^-7^ |
| chr12 | Z73 Problems related to life-management difficulty | 4.52 [2.41-8.47] | 2.56×10^-6^ |
| chr12 | D47 Other neoplasms of uncertain or unknown behaviour of lymphoid, haematopoietic and related tissue | 4.46 [2.38-8.35] | 3.11×10^-6^ |
| chr12 | G13 Systemic atrophies primarily affecting central nervous system in diseases classified elsewhere | 30.42 [7.04-131.4] | 4.75×10^-6^ |
| chr12 | D61 Other aplastic anaemias | 3.99 [2.19-7.27] | 5.87×10^-6^ |
| chr12 | M88 Paget's disease of bone [osteitis deformans] | 9.33 [3.43-25.39] | 1.22×10^-5^ |
| chr12 | A08 Viral and other specified intestinal infections | 2.56 [1.66-3.95] | 2.19×10^-5^ |
| chr12 | N16 Renal tubulo-interstitial disorders in diseases classified elsewhere | 21.42 [5.11-89.77] | 2.76×10^-5^ |
| chr12 | Y43 Primarily systemic agents | 2.65 [1.68-4.18] | 2.78×10^-5^ |
| chr13 | C91 Lymphoid leukaemia | 106.67 [87.43-130.14] | 0 |
| chr13 | D80 Immunodeficiency with predominantly antibody defects | 27.49 [16.87-44.77] | 2.03×10^-40^ |
| chr13 | D70 Agranulocytosis | 3.64 [2.73-4.86] | 1.47×10^-18^ |
| chr13 | C95 Leukaemia of unspecified cell type | 42.56 [17.91-101.18] | 2.05×10^-17^ |
| chr13 | D72 Other disorders of white blood cells | 7.16 [4.4-11.63] | 1.97×10^-15^ |
| chr13 | C83 Diffuse non-Hodgkin's lymphoma | 6.27 [3.96-9.93] | 4.93×10^-15^ |
| chr13 | C85 Other and unspecified types of non-Hodgkin's lymphoma | 5.99 [3.69-9.73] | 4.66×10^-13^ |
| chr13 | R16 Hepatomegaly and splenomegaly, not elsewhere classified | 6.88 [3.95-11.99] | 9.20×10^-12^ |
| chr13 | Z85 Personal history of malignant neoplasm | 1.89 [1.57-2.29] | 4.57×10^-11^ |
| chr13 | A41 Other septicaemia | 2.09 [1.64-2.67] | 3.32×10^-9^ |
| chr13 | Z29 Need for other prophylactic measures | 6.17 [3.29-11.59] | 1.51×10^-8^ |
| chr13 | D61 Other aplastic anaemias | 4.02 [2.26-7.14] | 2.06×10^-6^ |
| chr13 | Y43 Primarily systemic agents | 2.78 [1.8-4.3] | 3.79×10^-6^ |
| chr13 | Z51 Other medical care | 1.56 [1.27-1.9] | 1.36×10^-5^ |
| chr14 | C91 Lymphoid leukaemia | 18.42 [13.22-25.66] | 1.80×10^-66^ |
| chr14 | D70 Agranulocytosis | 2.62 [1.91-3.6] | 2.37×10^-9^ |
| chr14 | D46 Myelodysplastic syndromes | 7.5 [3.84-14.64] | 3.45×10^-9^ |
| chr14 | C83 Diffuse non-Hodgkin's lymphoma | 4.42 [2.64-7.4] | 1.49×10^-8^ |
| chr14 | D47 Other neoplasms of uncertain or unknown behaviour of lymphoid, haematopoietic and related tissue | 4.53 [2.55-8.04] | 2.56×10^-7^ |
| chr14 | D69 Purpura and other haemorrhagic conditions | 2.6 [1.8-3.77] | 4.29×10^-7^ |
| chr14 | C85 Other and unspecified types of non-Hodgkin's lymphoma | 3.78 [2.13-6.71] | 5.54×10^-6^ |
| chr14 | C88 Malignant immunoproliferative diseases | 9.12 [3.35-24.83] | 1.53×10^-5^ |
| chr15 | D84 Other immunodeficiencies | 21.58 [7.83-59.51] | 2.93×10^-9^ |
| chr15 | T21 Burn and corrosion of trunk | 19.17 [5.97-61.51] | 6.89×10^-7^ |
| chr15 | C85 Other and unspecified types of non-Hodgkin's lymphoma | 4.09 [2.18-7.67] | 1.12×10^-5^ |
| chr15 | C91 Lymphoid leukaemia | 4.4 [2.18-8.88] | 3.61×10^-5^ |
| chr16 | C82 Follicular [nodular] non-Hodgkin's lymphoma | 10.34 [4.87-21.95] | 1.21×10^-9^ |
| chr16 | I23 Certain current complications following acute myocardial infarction | 38.31 [11.74-125] | 1.52×10^-9^ |
| chr16 | C83 Diffuse non-Hodgkin's lymphoma | 5.56 [2.96-10.42] | 8.98×10^-8^ |
| chr16 | C91 Lymphoid leukaemia | 6.5 [3.22-13.12] | 1.79×10^-7^ |
| chr16 | A41 Other septicaemia | 2.14 [1.57-2.92] | 1.51×10^-6^ |
| chr16 | Z94 Transplanted organ and tissue status | 5.38 [2.55-11.37] | 1.05×10^-5^ |
| chr16 | F32 Depressive episode | 1.89 [1.41-2.53] | 2.02×10^-5^ |
| chr16 | H95 Postprocedural disorders of ear and mastoid process, not elsewhere classified | 21.27 [5.15-87.78] | 2.36×10^-5^ |
| chr16 | C92 Myeloid leukaemia | 6.44 [2.65-15.63] | 3.85×10^-5^ |
| chr17 | C91 Lymphoid leukaemia | 9.76 [5.71-16.69] | 7.91×10^-17^ |
| chr17 | C83 Diffuse non-Hodgkin's lymphoma | 7.64 [4.63-12.6] | 1.69×10^-15^ |
| chr17 | C85 Other and unspecified types of non-Hodgkin's lymphoma | 7.15 [4.19-12.19] | 5.10×10^-13^ |
| chr17 | C84 Peripheral and cutaneous T-cell lymphomas | 17.59 [7.73-40.01] | 8.08×10^-12^ |
| chr17 | Y43 Primarily systemic agents | 3.65 [2.34-5.7] | 1.27×10^-8^ |
| chr17 | D70 Agranulocytosis | 2.91 [2.01-4.22] | 1.80×10^-8^ |
| chr17 | D69 Purpura and other haemorrhagic conditions | 3.13 [2.04-4.79] | 1.52×10^-7^ |
| chr17 | C92 Myeloid leukaemia | 6.71 [2.98-15.11] | 4.20×10^-6^ |
| chr17 | L41 Parapsoriasis | 22.54 [5.4-93.99] | 1.91×10^-5^ |
| chr17 | E13 Other specified diabetes mellitus | 6.86 [2.83-16.66] | 2.08×10^-5^ |
| chr18 | C83 Diffuse non-Hodgkin's lymphoma | 16.68 [10.46-26.59] | 2.98×10^-32^ |
| chr18 | C85 Other and unspecified types of non-Hodgkin's lymphoma | 15.82 [9.68-25.85] | 3.30×10^-28^ |
| chr18 | C91 Lymphoid leukaemia | 15.26 [8.53-27.29] | 4.10×10^-20^ |
| chr18 | D70 Agranulocytosis | 4.22 [2.74-6.48] | 5.29×10^-11^ |
| chr18 | C82 Follicular [nodular] non-Hodgkin's lymphoma | 11.87 [4.88-28.91] | 5.03×10^-8^ |
| chr18 | C88 Malignant immunoproliferative diseases | 18.9 [5.96-59.94] | 6.01×10^-7^ |
| chr18 | D47 Other neoplasms of uncertain or unknown behaviour of lymphoid, haematopoietic and related tissue | 6.52 [2.9-14.65] | 5.76×10^-6^ |
| chr18 | D72 Other disorders of white blood cells | 6.38 [2.84-14.34] | 7.32×10^-6^ |
| chr18 | R16 Hepatomegaly and splenomegaly, not elsewhere classified | 6.79 [2.8-16.48] | 2.31×10^-5^ |
| chr19 | C85 Other and unspecified types of non-Hodgkin's lymphoma | 8.75 [4.91-15.57] | 1.71×10^-13^ |
| chr19 | C91 Lymphoid leukaemia | 8.85 [4.55-17.22] | 1.29×10^-10^ |
| chr19 | D70 Agranulocytosis | 3.28 [2.16-4.98] | 2.54×10^-8^ |
| chr19 | R16 Hepatomegaly and splenomegaly, not elsewhere classified | 7.33 [3.46-15.52] | 2.00×10^-7^ |
| chr19 | G03 Meningitis due to other and unspecified causes | 17.98 [5.68-56.89] | 8.87×10^-7^ |
| chr19 | C04 Malignant neoplasm of floor of mouth | 33.61 [7.83-144.32] | 2.27×10^-6^ |
| chr20 | D46 Myelodysplastic syndromes | 10.34 [4.85-22.04] | 1.46×10^-9^ |
| chr20 | C08 Malignant neoplasm of other and unspecified major salivary glands | 53.89 [12.58-230.81] | 7.78×10^-8^ |
| chr20 | C91 Lymphoid leukaemia | 6.15 [3.04-12.41] | 4.16×10^-7^ |
| chr20 | R16 Hepatomegaly and splenomegaly, not elsewhere classified | 5.96 [2.81-12.62] | 3.13×10^-6^ |
| chr20 | D45 Polycythaemia vera | 10.06 [3.72-27.24] | 5.53×10^-6^ |
| chr20 | D75 Other diseases of blood and blood-forming organs | 4.41 [2.27-8.54] | 1.12×10^-5^ |
| chr20 | B50 Plasmodium falciparum malaria | 22.36 [5.41-92.35] | 1.76×10^-5^ |
| chr20 | C07 Malignant neoplasm of parotid gland | 20.33 [4.93-83.77] | 3.06×10^-5^ |
| chr20 | J13 Pneumonia due to Streptococcus pneumoniae | 6.58 [2.71-15.99] | 3.16×10^-5^ |
| chr20 | D61 Other aplastic anaemias | 4.37 [2.16-8.81] | 3.83×10^-5^ |
| chr20 | D47 Other neoplasms of uncertain or unknown behaviour of lymphoid, haematopoietic and related tissue | 4.83 [2.28-10.21] | 3.83×10^-5^ |
| chr21 | C92 Myeloid leukaemia | 10.88 [4.82-24.55] | 8.85×10^-9^ |
| chr22 | C91 Lymphoid leukaemia | 22.85 [16.25-32.12] | 1.83×10^-72^ |
| chr22 | C85 Other and unspecified types of non-Hodgkin's lymphoma | 4.44 [2.44-8.09] | 1.05×10^-6^ |
| chr22 | Z29 Need for other prophylactic measures | 5.64 [2.8-11.39] | 1.37×10^-6^ |
| chr22 | D70 Agranulocytosis | 2.45 [1.7-3.53] | 1.44×10^-6^ |
| chr22 | Y41 Other systemic anti-infectives and antiparasitics | 9.55 [3.53-25.88] | 9.08×10^-6^ |
| chr22 | C94 Other leukaemias of specified cell type | 25.54 [6.05-107.81] | 1.04×10^-5^ |
| chr22 | A02 Other Salmonella infections | 11.93 [3.77-37.77] | 2.48×10^-5^ |
| chr22 | D59 Acquired haemolytic anaemia | 8.17 [3.02-22.12] | 3.53×10^-5^ |

**Table S4.** Number of autosomal mCAs with statistically significant association with incident disease risk adjusted for age, age^2^, smoking, genetic ancestry, and sex (only for autosomal mCA analyses).

| Disease | Autosomes involved | Number of cases |
| --- | --- | --- |
| C91 Lymphoid leukaemia | 21 | 616 |
| C85 Other and unspecified types of non-Hodgkin's lymphoma | 17 | 859 |
| D70 Agranulocytosis | 16 | 4637 |
| C83 Diffuse non-Hodgkin's lymphoma | 14 | 903 |
| R16 Hepatomegaly and splenomegaly, not elsewhere classified | 10 | 611 |
| C92 Myeloid leukaemia | 9 | 381 |
| D47 Other neoplasms of uncertain or unknown behaviour of lymphoid, haematopoietic and related tissue | 9 | 729 |
| A41 Other septicaemia | 8 | 10144 |
| C82 Follicular [nodular] non-Hodgkin's lymphoma | 8 | 347 |
| D46 Myelodysplastic syndromes | 8 | 302 |
| D61 Other aplastic anaemias | 8 | 879 |
| D69 Purpura and other haemorrhagic conditions | 6 | 3090 |
| D80 Immunodeficiency with predominantly antibody defects | 6 | 248 |
| Y43 Primarily systemic agents | 6 | 2494 |
| Z51 Other medical care | 6 | 23428 |
| C88 Malignant immunoproliferative diseases | 5 | 121 |
| Z94 Transplanted organ and tissue status | 5 | 724 |
| B25 Cytomegaloviral disease | 4 | 171 |
| C84 Peripheral and cutaneous T-cell lymphomas | 4 | 154 |
| C95 Leukaemia of unspecified cell type | 3 | 50 |
| D72 Other disorders of white blood cells | 3 | 794 |
| R50 Fever of unknown origin | 3 | 3504 |
| T86 Failure and rejection of transplanted organs and tissues | 3 | 232 |
| C94 Other leukaemias of specified cell type | 2 | 32 |
| D45 Polycythaemia vera | 2 | 201 |
| D58 Other hereditary haemolytic anaemias | 2 | 162 |
| D75 Other diseases of blood and blood-forming organs | 2 | 1059 |
| D84 Other immunodeficiencies | 2 | 93 |
| E13 Other specified diabetes mellitus | 2 | 349 |
| J18 Pneumonia, organism unspecified | 2 | 17638 |
| J90 Pleural effusion, not elsewhere classified | 2 | 10097 |
| Z29 Need for other prophylactic measures | 2 | 527 |
| A02 Other Salmonella infections | 1 | 111 |
| A08 Viral and other specified intestinal infections | 1 | 3110 |
| B44 Aspergillosis | 1 | 259 |
| B50 Plasmodium falciparum malaria | 1 | 68 |
| B82 Unspecified intestinal parasitism | 1 | 46 |
| B96 Other bacterial agents as the cause of diseases classified to other chapters | 1 | 9917 |
| C04 Malignant neoplasm of floor of mouth | 1 | 39 |
| C07 Malignant neoplasm of parotid gland | 1 | 60 |
| C34 Malignant neoplasm of bronchus and lung | 1 | 2801 |
| C69 Malignant neoplasm of eye and adnexa | 1 | 96 |
| C75 Malignant neoplasm of other endocrine glands and related structures | 1 | 48 |
| C86 Other specified types of T/NK-cell lymphoma | 1 | 33 |
| D59 Acquired haemolytic anaemia | 1 | 177 |
| D64 Other anaemias | 1 | 16970 |
| D65 Disseminated intravascular coagulation [defibrination syndrome] | 1 | 70 |
| D73 Diseases of spleen | 1 | 767 |
| E83 Disorders of mineral metabolism | 1 | 5293 |
| F32 Depressive episode | 1 | 14883 |

**Table S5.** Statistically significant associations between mCAs and incident disease risk adjusted for age, age^2^, smoking, and sex (only for autosomal mCA) restricted to participants without cancer diagnoses prior to enrollment.

| mCAs | Disease | Odds Ratio [95% Confidence Interval] | P-value |
| --- | --- | --- | --- |
| Autosome | C91 Lymphoid leukaemia | 21.82 [19.83-24.02] | 7.42×10^-227^ |
| Autosome | D45 Polycythaemia vera | 14.16 [12.02-16.69] | 1.07×10^-58^ |
| Autosome | D47 Other neoplasms of uncertain or unknown behaviour of lymphoid, haematopoietic and related tissue | 5.43 [4.86-6.06] | 4.77×10^-53^ |
| Autosome | C83 Diffuse non-Hodgkin's lymphoma | 4.33 [3.9-4.81] | 6.13×10^-44^ |
| Autosome | D70 Agranulocytosis | 2.23 [2.09-2.38] | 3.09×10^-36^ |
| Autosome | D46 Myelodysplastic syndromes | 6.64 [5.66-7.78] | 8.86×10^-33^ |
| Autosome | C85 Other and unspecified types of non-Hodgkin's lymphoma | 3.88 [3.46-4.35] | 4.75×10^-32^ |
| Autosome | C92 Myeloid leukaemia | 4.54 [3.86-5.33] | 6.20×10^-21^ |
| Autosome | R16 Hepatomegaly and splenomegaly, not elsewhere classified | 3.48 [3.01-4.01] | 2.56×10^-18^ |
| Autosome | D69 Purpura and other haemorrhagic conditions | 1.93 [1.78-2.08] | 5.84×10^-17^ |
| Autosome | A41 Other septicaemia | 1.48 [1.41-1.55] | 2.81×10^-15^ |
| Autosome | D75 Other diseases of blood and blood-forming organs | 2.53 [2.24-2.85] | 1.46×10^-14^ |
| Autosome | C95 Leukaemia of unspecified cell type | 14.97 [9.99-22.44] | 2.29×10^-11^ |
| Autosome | D80 Immunodeficiency with predominantly antibody defects | 4.08 [3.29-5.07] | 8.90×10^-11^ |
| Autosome | D72 Other disorders of white blood cells | 2.48 [2.15-2.86] | 1.17×10^-10^ |
| Autosome | C84 Peripheral and cutaneous T-cell lymphomas | 4.95 [3.81-6.42] | 8.75×10^-10^ |
| Autosome | J18 Pneumonia, organism unspecified | 1.27 [1.22-1.33] | 1.11×10^-9^ |
| Autosome | C93 Monocytic leukaemia | 6.82 [4.8-9.7] | 4.75×10^-8^ |
| Autosome | D61 Other aplastic anaemias | 2.15 [1.87-2.48] | 5.01×10^-8^ |
| Autosome | C82 Follicular [nodular] non-Hodgkin's lymphoma | 2.76 [2.27-3.36] | 2.65×10^-7^ |
| Autosome | Z51 Other medical care | 1.2 [1.16-1.25] | 5.28×10^-7^ |
| Autosome | C94 Other leukaemias of specified cell type | 11.44 [6.96-18.79] | 9.23×10^-7^ |
| Autosome | Y43 Primarily systemic agents | 1.62 [1.46-1.79] | 1.35×10^-6^ |
| Autosome | J98 Other respiratory disorders | 1.25 [1.19-1.31] | 2.85×10^-6^ |
| Autosome | C88 Malignant immunoproliferative diseases | 4.1 [3.03-5.54] | 3.13×10^-6^ |
| Autosome | N17 Acute renal failure | 1.22 [1.16-1.27] | 8.93×10^-6^ |
| Autosome | D59 Acquired haemolytic anaemia | 3.24 [2.48-4.23] | 1.16×10^-5^ |
| mLOX | J03 Acute tonsillitis | 1.84 [1.6-2.11] | 9.18×10^-6^ |
| mLOY | E11 Non-insulin-dependent diabetes mellitus | 0.79 [0.77-0.81] | 7.04×10^-22^ |
| mLOY | I10 Essential (primary) hypertension | 0.9 [0.89-0.92] | 2.26×10^-12^ |
| mLOY | H36 Retinal disorders in diseases classified elsewhere | 0.62 [0.57-0.67] | 1.41×10^-9^ |
| mLOY | E66 Obesity | 0.85 [0.83-0.88] | 4.73×10^-9^ |
| mLOY | E14 Unspecified diabetes mellitus | 0.68 [0.63-0.73] | 2.60×10^-7^ |
| mLOY | G63 Polyneuropathy in diseases classified elsewhere | 0.61 [0.54-0.68] | 1.31×10^-5^ |

**Table S6.** Statistically significant associations between mCAs and incident disease risk adjusted for age, age^2^, smoking, body mass index (BMI), and sex (only for autosomal mCA analyses).

| mCAs | Disease | Odds Ratio [95% Confidence Interval] | P-value |
| --- | --- | --- | --- |
| Autosome | C91 Lymphoid leukaemia | 24.08 [20.47, 28.34] | <5×10^-324^ |
| Autosome | D47 Other neoplasms of uncertain or unknown behaviour of lymphoid, haematopoietic and related tissue | 5.38 [4.45, 6.51] | 3.95×10^-67^ |
| Autosome | C83 Diffuse non-Hodgkin's lymphoma | 4.68 [3.92, 5.59] | 6.51×10^-66^ |
| Autosome | D45 Polycythaemia vera | 12.33 [9.14, 16.64] | 1.39×10^-60^ |
| Autosome | C85 Other and unspecified types of non-Hodgkin's lymphoma | 4.35 [3.61, 5.24] | 2.59×10^-54^ |
| Autosome | D70 Agranulocytosis | 2.23 [2, 2.48] | 1.08×10^-48^ |
| Autosome | D46 Myelodysplastic syndromes | 6.09 [4.62, 8.04] | 2.20×10^-37^ |
| Autosome | C92 Myeloid leukaemia | 5.14 [3.95, 6.7] | 7.58×10^-34^ |
| Autosome | R16 Hepatomegaly and splenomegaly, not elsewhere classified | 3.88 [3.06, 4.92] | 3.95×10^-29^ |
| Autosome | A41 Other septicaemia | 1.55 [1.42, 1.68] | 1.05×10^-24^ |
| Autosome | D80 Immunodeficiency with predominantly antibody defects | 5.65 [4.06, 7.86] | 1.05×10^-24^ |
| Autosome | D69 Purpura and other haemorrhagic conditions | 2 [1.75, 2.29] | 2.77×10^-24^ |
| Autosome | D75 Other diseases of blood and blood-forming organs | 2.79 [2.28, 3.42] | 2.74×10^-23^ |
| Autosome | C95 Leukaemia of unspecified cell type | 13.42 [7.46, 24.14] | 4.48×10^-18^ |
| Autosome | D72 Other disorders of white blood cells | 2.71 [2.14, 3.43] | 1.29×10^-16^ |
| Autosome | J18 Pneumonia, organism unspecified | 1.33 [1.24, 1.42] | 2.54×10^-16^ |
| Autosome | C94 Other leukaemias of specified cell type | 14.06 [6.65, 29.74] | 4.61×10^-12^ |
| Autosome | D61 Other aplastic anaemias | 2.27 [1.8, 2.87] | 6.00×10^-12^ |
| Autosome | C84 Peripheral and cutaneous T-cell lymphomas | 4.45 [2.89, 6.86] | 1.30×10^-11^ |
| Autosome | Z51 Other medical care | 1.24 [1.16, 1.32] | 5.51×10^-11^ |
| Autosome | C88 Malignant immunoproliferative diseases | 4.26 [2.6, 6.99] | 9.25×10^-9^ |
| Autosome | Y43 Primarily systemic agents | 1.61 [1.37, 1.9] | 1.39×10^-8^ |
| Autosome | Z85 Personal history of malignant neoplasm | 1.21 [1.13, 1.29] | 2.23×10^-8^ |
| Autosome | C93 Monocytic leukaemia | 6.3 [3.26, 12.15] | 4.13×10^-8^ |
| Autosome | C82 Follicular [nodular] non-Hodgkin's lymphoma | 2.67 [1.88, 3.8] | 4.46×10^-8^ |
| Autosome | B25 Cytomegaloviral disease | 3.7 [2.31, 5.93] | 4.96×10^-8^ |
| Autosome | J90 Pleural effusion, not elsewhere classified | 1.27 [1.17, 1.39] | 9.39×10^-8^ |
| Autosome | J98 Other respiratory disorders | 1.25 [1.15, 1.36] | 1.39×10^-7^ |
| Autosome | D64 Other anaemias | 1.21 [1.12, 1.3] | 3.96×10^-7^ |
| Autosome | Z94 Transplanted organ and tissue status | 2.05 [1.54, 2.72] | 7.88×10^-7^ |
| Autosome | E83 Disorders of mineral metabolism | 3.06 [1.94, 4.82] | 1.41×10^-6^ |
| Autosome | D59 Acquired haemolytic anaemia | 3.09 [1.93, 4.94] | 2.47×10^-6^ |
| Autosome | R50 Fever of unknown origin | 1.41 [1.22, 1.64] | 6.51×10^-6^ |
| Autosome | T86 Failure and rejection of transplanted organs and tissues | 2.79 [1.77, 4.38] | 9.14×10^-6^ |
| Autosome | R60 Oedema, not elsewhere classified | 1.5 [1.25, 1.8] | 1.51×10^-5^ |
| Autosome | R22 Localised swelling, mass and lump of skin and subcutaneous tissue | 1.67 [1.31, 2.13] | 3.11×10^-5^ |
| mLOX | C91 Lymphoid leukaemia | 2.5 [1.74, 3.61] | 8.36×10^-7^ |
| mLOX | J03 Acute tonsillitis | 1.82 [1.43, 2.34] | 1.83×10^-6^ |
| mLOY | H36 Retinal disorders in diseases classified elsewhere | 0.67 [0.58, 0.77] | 6.36×10^-8^ |
| mLOY | I10 Essential (primary) hypertension | 0.94 [0.91, 0.96] | 1.35×10^-6^ |

**Table S7.** Statistically significant associations between prevalent disease diagnoses and mCAs adjusted for age, age^2^, smoking, and sex (only for autosomal mCA analyses).

| mCAs | Disease | Odds Ratio [95% Confidence Interval] | P-value |
| --- | --- | --- | --- |
| Autosome | C91 Lymphoid leukaemia | 27.75 [19.69, 39.09] | 1.76×10^-80^ |
| Autosome | D47 Other neoplasms of uncertain or unknown behaviour of lymphoid, haematopoietic and related tissue | 10.56 [7.18, 15.52] | 3.65×10^-33^ |
| Autosome | D45 Polycythaemia vera | 6.87 [4.76, 9.9] | 5.56×10^-25^ |
| Autosome | Z37 Outcome of delivery | 0.52 [0.46, 0.59] | 3.85×10^-23^ |
| Autosome | I10 Essential (primary) hypertension | 1.26 [1.2, 1.32] | 3.67×10^-21^ |
| Autosome | E78 Disorders of lipoprotein metabolism and other lipidaemias | 1.21 [1.16, 1.26] | 2.28×10^-19^ |
| Autosome | C85 Other and unspecified types of non-Hodgkin's lymphoma | 4.09 [2.93, 5.7] | 1.11×10^-16^ |
| Autosome | M19 Other arthrosis | 1.22 [1.16, 1.28] | 1.05×10^-15^ |
| Autosome | N40 Hyperplasia of prostate | 1.37 [1.26, 1.49] | 2.94×10^-13^ |
| Autosome | D46 Myelodysplastic syndromes | 10.42 [5.5, 19.74] | 6.76×10^-13^ |
| Autosome | N92 Excessive, frequent and irregular menstruation | 0.74 [0.68, 0.81] | 1.87×10^-12^ |
| Autosome | I25 Chronic ischaemic heart disease | 1.32 [1.22, 1.43] | 5.20×10^-12^ |
| Autosome | I20 Angina pectoris | 1.29 [1.2, 1.39] | 5.93×10^-12^ |
| Autosome | I21 Acute myocardial infarction | 1.38 [1.26, 1.51] | 7.69×10^-12^ |
| Autosome | C83 Diffuse non-Hodgkin's lymphoma | 4.66 [2.9, 7.49] | 2.08×10^-10^ |
| Autosome | Z30 Contraceptive management | 0.66 [0.57, 0.76] | 3.25×10^-9^ |
| Autosome | C61 Malignant neoplasm of prostate | 1.59 [1.36, 1.85] | 6.69×10^-9^ |
| Autosome | C44 Other malignant neoplasms of skin | 1.32 [1.2, 1.46] | 2.63×10^-8^ |
| Autosome | M10 Gout | 1.31 [1.19, 1.45] | 4.50×10^-8^ |
| Autosome | D59 Acquired haemolytic anaemia | 4.83 [2.73, 8.52] | 5.72×10^-8^ |
| Autosome | L57 Skin changes due to chronic exposure to nonionising radiation | 1.37 [1.22, 1.54] | 7.41×10^-8^ |
| Autosome | H26 Other cataract | 1.28 [1.17, 1.4] | 9.27×10^-8^ |
| Autosome | Z85 Personal history of malignant neoplasm | 1.3 [1.18, 1.43] | 1.10×10^-7^ |
| Autosome | O80 Single spontaneous delivery | 0.75 [0.68, 0.84] | 1.59×10^-7^ |
| Autosome | Z95 Presence of cardiac and vascular implants and grafts | 1.39 [1.23, 1.58] | 1.74×10^-7^ |
| Autosome | K57 Diverticular disease of intestine | 1.23 [1.13, 1.33] | 1.07×10^-6^ |
| Autosome | M81 Osteoporosis without pathological fracture | 1.29 [1.16, 1.43] | 1.50×10^-6^ |
| Autosome | I48 Atrial fibrillation and flutter | 1.31 [1.17, 1.46] | 1.56×10^-6^ |
| Autosome | K40 Inguinal hernia | 1.23 [1.13, 1.33] | 1.69×10^-6^ |
| Autosome | H40 Glaucoma | 1.32 [1.17, 1.48] | 2.58×10^-6^ |
| Autosome | M16 Coxarthrosis [arthrosis of hip] | 1.33 [1.18, 1.5] | 2.69×10^-6^ |
| Autosome | O26 Maternal care for other conditions predominantly related to pregnancy | 0.46 [0.33, 0.64] | 4.39×10^-6^ |
| Autosome | R16 Hepatomegaly and splenomegaly, not elsewhere classified | 2.86 [1.82, 4.49] | 4.66×10^-6^ |
| Autosome | D75 Other diseases of blood and blood-forming organs | 1.88 [1.43, 2.47] | 5.99×10^-6^ |
| Autosome | M47 Spondylosis | 1.2 [1.11, 1.31] | 6.98×10^-6^ |
| Autosome | Z51 Other medical care | 1.32 [1.17, 1.49] | 1.19×10^-5^ |
| Autosome | Z08 Follow-up examination after treatment for malignant neoplasm | 1.48 [1.24, 1.77] | 1.35×10^-5^ |
| Autosome | J30 Vasomotor and allergic rhinitis | 0.88 [0.83, 0.93] | 2.68×10^-5^ |
| Autosome | I70 Atherosclerosis | 1.98 [1.43, 2.73] | 3.15×10^-5^ |
| mLOX | Z37 Outcome of delivery | 0.26 [0.22, 0.3] | 6.33×10^-67^ |
| mLOX | M19 Other arthrosis | 1.39 [1.32, 1.46] | 4.03×10^-36^ |
| mLOX | E78 Disorders of lipoprotein metabolism and other lipidaemias | 1.39 [1.32, 1.47] | 5.91×10^-36^ |
| mLOX | I10 Essential (primary) hypertension | 1.43 [1.35, 1.52] | 3.66×10^-34^ |
| mLOX | N95 Menopausal and other perimenopausal disorders | 1.32 [1.25, 1.41] | 9.61×10^-21^ |
| mLOX | K44 Diaphragmatic hernia | 1.41 [1.3, 1.52] | 4.55×10^-18^ |
| mLOX | Z30 Contraceptive management | 0.4 [0.32, 0.49] | 4.73×10^-18^ |
| mLOX | I20 Angina pectoris | 1.55 [1.41, 1.72] | 7.52×10^-18^ |
| mLOX | H26 Other cataract | 1.54 [1.39, 1.7] | 2.20×10^-17^ |
| mLOX | C44 Other malignant neoplasms of skin | 1.59 [1.42, 1.78] | 2.17×10^-16^ |
| mLOX | M47 Spondylosis | 1.41 [1.3, 1.54] | 3.68×10^-15^ |
| mLOX | K57 Diverticular disease of intestine | 1.43 [1.3, 1.56] | 1.28×10^-14^ |
| mLOX | I25 Chronic ischaemic heart disease | 1.64 [1.45, 1.87] | 2.91×10^-14^ |
| mLOX | N92 Excessive, frequent and irregular menstruation | 0.76 [0.71, 0.82] | 2.11×10^-13^ |
| mLOX | N81 Female genital prolapse | 1.38 [1.26, 1.51] | 3.25×10^-12^ |
| mLOX | O26 Maternal care for other conditions predominantly related to pregnancy | 0.28 [0.19, 0.4] | 5.39×10^-12^ |
| mLOX | M81 Osteoporosis without pathological fracture | 1.39 [1.26, 1.53] | 1.19×10^-11^ |
| mLOX | I21 Acute myocardial infarction | 1.75 [1.49, 2.06] | 1.64×10^-11^ |
| mLOX | M17 Gonarthrosis [arthrosis of knee] | 1.41 [1.27, 1.56] | 3.75×10^-11^ |
| mLOX | K80 Cholelithiasis | 1.3 [1.2, 1.41] | 1.09×10^-10^ |
| mLOX | I48 Atrial fibrillation and flutter | 1.66 [1.41, 1.94] | 3.70×10^-10^ |
| mLOX | O03 Spontaneous abortion | 0.59 [0.5, 0.7] | 7.82×10^-10^ |
| mLOX | K29 Gastritis and duodenitis | 1.29 [1.19, 1.4] | 1.92×10^-9^ |
| mLOX | M16 Coxarthrosis [arthrosis of hip] | 1.5 [1.32, 1.72] | 2.17×10^-9^ |
| mLOX | M79 Other soft tissue disorders, not elsewhere classified | 1.19 [1.12, 1.26] | 4.04×10^-9^ |
| mLOX | H61 Other disorders of external ear | 1.36 [1.22, 1.5] | 4.60×10^-9^ |
| mLOX | O34 Maternal care for known or suspected abnormality of pelvic organs | 0.33 [0.23, 0.48] | 5.56×10^-9^ |
| mLOX | O32 Maternal care for known or suspected malpresentation of foetus | 0.2 [0.11, 0.34] | 6.11×10^-9^ |
| mLOX | K20 Oesophagitis | 1.54 [1.33, 1.78] | 8.45×10^-9^ |
| mLOX | H40 Glaucoma | 1.48 [1.29, 1.7] | 1.61×10^-8^ |
| mLOX | K21 Gastro-oesophageal reflux disease | 1.22 [1.14, 1.3] | 1.63×10^-8^ |
| mLOX | L98 Other disorders of skin and subcutaneous tissue, not elsewhere classified | 1.25 [1.16, 1.35] | 1.85×10^-8^ |
| mLOX | N97 Female infertility | 0.56 [0.46, 0.69] | 2.35×10^-8^ |
| mLOX | N94 Pain and other conditions associated with female genital organs and menstrual cycle | 0.73 [0.66, 0.82] | 5.02×10^-8^ |
| mLOX | J30 Vasomotor and allergic rhinitis | 0.82 [0.77, 0.89] | 1.35×10^-7^ |
| mLOX | O80 Single spontaneous delivery | 0.79 [0.72, 0.86] | 1.40×10^-7^ |
| mLOX | M20 Acquired deformities of fingers and toes | 1.28 [1.16, 1.4] | 3.09×10^-7^ |
| mLOX | O02 Other abnormal products of conception | 0.44 [0.32, 0.6] | 4.79×10^-7^ |
| mLOX | L57 Skin changes due to chronic exposure to nonionising radiation | 1.42 [1.24, 1.64] | 7.57×10^-7^ |
| mLOX | I80 Phlebitis and thrombophlebitis | 1.3 [1.17, 1.44] | 9.07×10^-7^ |
| mLOX | N18 Chronic renal failure | 1.46 [1.25, 1.7] | 1.05×10^-6^ |
| mLOX | Z96 Presence of other functional implants | 1.48 [1.26, 1.73] | 1.30×10^-6^ |
| mLOX | Z92 Personal history of medical treatment | 1.39 [1.21, 1.59] | 1.70×10^-6^ |
| mLOX | D12 Benign neoplasm of colon, rectum, anus and anal canal | 1.49 [1.26, 1.75] | 1.77×10^-6^ |
| mLOX | O20 Haemorrhage in early pregnancy | 0.38 [0.26, 0.57] | 2.17×10^-6^ |
| mLOX | T84 Complications of internal orthopaedic prosthetic devices, implants and grafts | 1.68 [1.35, 2.09] | 2.45×10^-6^ |
| mLOX | H04 Disorders of lachrymal system | 1.35 [1.19, 1.53] | 2.79×10^-6^ |
| mLOX | N73 Other female pelvic inflammatory diseases | 0.6 [0.49, 0.75] | 5.11×10^-6^ |
| mLOX | K74 Fibrosis and cirrhosis of liver | 2.27 [1.59, 3.24] | 6.12×10^-6^ |
| mLOX | O60 Preterm delivery | 0.24 [0.13, 0.46] | 9.30×10^-6^ |
| mLOX | J44 Other chronic obstructive pulmonary disease | 1.48 [1.24, 1.76] | 9.99×10^-6^ |
| mLOX | N80 Endometriosis | 0.76 [0.67, 0.86] | 1.07×10^-5^ |
| mLOX | Z82 Family history of certain disabilities and chronic diseases leading to disablement | 1.42 [1.21, 1.67] | 1.37×10^-5^ |
| mLOX | E03 Other hypothyroidism | 1.15 [1.08, 1.23] | 1.96×10^-5^ |
| mLOX | R31 Unspecified haematuria | 1.33 [1.17, 1.52] | 2.39×10^-5^ |
| mLOX | H43 Disorders of vitreous body | 1.41 [1.2, 1.65] | 2.99×10^-5^ |
| mLOX | K30 Dyspepsia | 1.28 [1.14, 1.44] | 3.00×10^-5^ |
| mLOX | N39 Other disorders of urinary system | 1.15 [1.08, 1.23] | 3.57×10^-5^ |
| mLOX | A15 Respiratory tuberculosis, bacteriologically and histologically confirmed | 1.56 [1.26, 1.92] | 3.75×10^-5^ |
| mLOX | L43 Lichen planus | 1.79 [1.35, 2.35] | 3.95×10^-5^ |
| mLOX | Z95 Presence of cardiac and vascular implants and grafts | 1.59 [1.27, 1.99] | 4.64×10^-5^ |
| mLOX | J22 Unspecified acute lower respiratory infection | 1.16 [1.08, 1.25] | 4.93×10^-5^ |
| mLOY | E78 Disorders of lipoprotein metabolism and other lipidaemias | 1.5 [1.46, 1.53] | 3.71×10^-205^ |
| mLOY | N40 Hyperplasia of prostate | 1.78 [1.71, 1.85] | 2.39×10^-172^ |
| mLOY | I25 Chronic ischaemic heart disease | 1.76 [1.69, 1.84] | 4.45×10^-149^ |
| mLOY | I20 Angina pectoris | 1.7 [1.63, 1.77] | 5.52×10^-142^ |
| mLOY | I10 Essential (primary) hypertension | 1.47 [1.42, 1.51] | 1.48×10^-129^ |
| mLOY | I21 Acute myocardial infarction | 1.79 [1.71, 1.88] | 2.78×10^-125^ |
| mLOY | F17 Mental and behavioural disorders due to use of tobacco | 1.63 [1.56, 1.71] | 5.30×10^-103^ |
| mLOY | Z72 Problems related to lifestyle | 1.79 [1.7, 1.9] | 6.24×10^-93^ |
| mLOY | M19 Other arthrosis | 1.46 [1.41, 1.51] | 1.37×10^-92^ |
| mLOY | Z30 Contraceptive management | 0.28 [0.25, 0.32] | 2.81×10^-88^ |
| mLOY | J44 Other chronic obstructive pulmonary disease | 2.3 [2.11, 2.51] | 1.20×10^-81^ |
| mLOY | K40 Inguinal hernia | 1.49 [1.43, 1.55] | 2.99×10^-81^ |
| mLOY | C44 Other malignant neoplasms of skin | 1.7 [1.6, 1.82] | 6.59×10^-60^ |
| mLOY | C61 Malignant neoplasm of prostate | 1.86 [1.73, 2.01] | 2.17×10^-56^ |
| mLOY | K57 Diverticular disease of intestine | 1.57 [1.48, 1.66] | 7.81×10^-56^ |
| mLOY | Z95 Presence of cardiac and vascular implants and grafts | 1.7 [1.59, 1.82] | 2.15×10^-55^ |
| mLOY | I48 Atrial fibrillation and flutter | 1.61 [1.51, 1.71] | 1.34×10^-51^ |
| mLOY | I73 Other peripheral vascular diseases | 1.98 [1.81, 2.17] | 5.83×10^-51^ |
| mLOY | I64 Stroke, not specified as haemorrhage or infarction | 1.72 [1.6, 1.85] | 9.23×10^-50^ |
| mLOY | H26 Other cataract | 1.6 [1.5, 1.7] | 1.54×10^-49^ |
| mLOY | Z86 Personal history of certain other diseases | 1.48 [1.4, 1.56] | 4.05×10^-49^ |
| mLOY | L57 Skin changes due to chronic exposure to nonionising radiation | 1.75 [1.63, 1.89] | 4.70×10^-48^ |
| mLOY | M47 Spondylosis | 1.48 [1.4, 1.57] | 1.37×10^-40^ |
| mLOY | G45 Transient cerebral ischaemic attacks and related syndromes | 1.82 [1.66, 2] | 4.38×10^-36^ |
| mLOY | D12 Benign neoplasm of colon, rectum, anus and anal canal | 1.64 [1.52, 1.78] | 4.33×10^-34^ |
| mLOY | Z92 Personal history of medical treatment | 1.54 [1.44, 1.65] | 1.80×10^-33^ |
| mLOY | Z85 Personal history of malignant neoplasm | 1.54 [1.43, 1.65] | 4.49×10^-32^ |
| mLOY | N32 Other disorders of bladder | 1.52 [1.41, 1.62] | 1.22×10^-31^ |
| mLOY | M16 Coxarthrosis [arthrosis of hip] | 1.64 [1.51, 1.79] | 3.36×10^-31^ |
| mLOY | J30 Vasomotor and allergic rhinitis | 0.79 [0.75, 0.82] | 2.34×10^-30^ |
| mLOY | K44 Diaphragmatic hernia | 1.32 [1.26, 1.39] | 1.58×10^-28^ |
| mLOY | L82 Seborrhoeic keratosis | 1.42 [1.33, 1.51] | 1.81×10^-27^ |
| mLOY | R79 Other abnormal findings of blood chemistry | 1.73 [1.57, 1.91] | 1.94×10^-27^ |
| mLOY | K26 Duodenal ulcer | 1.48 [1.38, 1.6] | 2.24×10^-26^ |
| mLOY | M17 Gonarthrosis [arthrosis of knee] | 1.36 [1.28, 1.44] | 3.01×10^-25^ |
| mLOY | H40 Glaucoma | 1.48 [1.38, 1.6] | 8.56×10^-25^ |
| mLOY | I71 Aortic aneurysm and dissection | 2.71 [2.24, 3.29] | 4.44×10^-24^ |
| mLOY | Z96 Presence of other functional implants | 1.6 [1.46, 1.76] | 1.98×10^-23^ |
| mLOY | I50 Heart failure | 1.67 [1.51, 1.85] | 1.14×10^-22^ |
| mLOY | R31 Unspecified haematuria | 1.37 [1.29, 1.46] | 2.16×10^-22^ |
| mLOY | R33 Retention of urine | 1.62 [1.47, 1.78] | 8.53×10^-22^ |
| mLOY | K29 Gastritis and duodenitis | 1.26 [1.2, 1.33] | 1.77×10^-21^ |
| mLOY | H25 Senile cataract | 1.81 [1.6, 2.05] | 3.24×10^-21^ |
| mLOY | J43 Emphysema | 2.53 [2.07, 3.09] | 6.17×10^-20^ |
| mLOY | M72 Fibroblastic disorders | 1.33 [1.25, 1.42] | 7.24×10^-20^ |
| mLOY | Z87 Personal history of other diseases and conditions | 1.35 [1.26, 1.44] | 9.79×10^-20^ |
| mLOY | M75 Shoulder lesions | 1.31 [1.24, 1.39] | 1.58×10^-19^ |
| mLOY | N18 Chronic renal failure | 1.5 [1.37, 1.63] | 2.57×10^-19^ |
| mLOY | Z09 Follow-up examination after treatment for conditions other than malignant neoplasms | 1.45 [1.34, 1.58] | 8.94×10^-19^ |
| mLOY | K80 Cholelithiasis | 1.41 [1.3, 1.52] | 1.89×10^-18^ |
| mLOY | M13 Other arthritis | 1.36 [1.27, 1.46] | 2.19×10^-18^ |
| mLOY | I70 Atherosclerosis | 2.37 [1.94, 2.89] | 1.36×10^-17^ |
| mLOY | H91 Other hearing loss | 1.48 [1.35, 1.62] | 1.55×10^-17^ |
| mLOY | I65 Occlusion and stenosis of precerebral arteries, not resulting in cerebral infarction | 2.65 [2.11, 3.32] | 2.24×10^-17^ |
| mLOY | K21 Gastro-oesophageal reflux disease | 1.19 [1.14, 1.23] | 4.42×10^-17^ |
| mLOY | Z53 Persons encountering health services for specifie procedures, not carried out | 1.28 [1.2, 1.35] | 1.83×10^-16^ |
| mLOY | I77 Other disorders of arteries and arterioles | 2.15 [1.79, 2.59] | 5.68×10^-16^ |
| mLOY | I83 Varicose veins of lower extremities | 1.32 [1.23, 1.41] | 1.18×10^-15^ |
| mLOY | H61 Other disorders of external ear | 1.24 [1.18, 1.31] | 1.75×10^-15^ |
| mLOY | K63 Other diseases of intestine | 1.4 [1.29, 1.52] | 4.23×10^-15^ |
| mLOY | Z82 Family history of certain disabilities and chronic diseases leading to disablement | 1.34 [1.24, 1.44] | 6.97×10^-15^ |
| mLOY | H43 Disorders of vitreous body | 1.57 [1.4, 1.76] | 8.81×10^-15^ |
| mLOY | A15 Respiratory tuberculosis, bacteriologically and histologically confirmed | 1.69 [1.48, 1.93] | 9.24×10^-15^ |
| mLOY | Z08 Follow-up examination after treatment for malignant neoplasm | 1.56 [1.4, 1.75] | 9.57×10^-15^ |
| mLOY | M81 Osteoporosis without pathological fracture | 1.61 [1.42, 1.82] | 5.60×10^-14^ |
| mLOY | R07 Pain in throat and chest | 1.2 [1.14, 1.26] | 7.70×10^-14^ |
| mLOY | N39 Other disorders of urinary system | 1.29 [1.21, 1.38] | 1.20×10^-13^ |
| mLOY | J22 Unspecified acute lower respiratory infection | 1.18 [1.13, 1.23] | 1.78×10^-13^ |
| mLOY | I49 Other cardiac arrhythmias | 1.42 [1.3, 1.56] | 1.80×10^-13^ |
| mLOY | E03 Other hypothyroidism | 1.33 [1.23, 1.44] | 2.99×10^-13^ |
| mLOY | I74 Arterial embolism and thrombosis | 2.24 [1.8, 2.79] | 4.04×10^-13^ |
| mLOY | C67 Malignant neoplasm of bladder | 1.75 [1.5, 2.04] | 5.36×10^-13^ |
| mLOY | M15 Polyarthrosis | 1.67 [1.45, 1.92] | 7.11×10^-13^ |
| mLOY | M79 Other soft tissue disorders, not elsewhere classified | 1.15 [1.11, 1.2] | 9.79×10^-13^ |
| mLOY | K25 Gastric ulcer | 1.33 [1.23, 1.45] | 2.57×10^-12^ |
| mLOY | G56 Mononeuropathies of upper limb | 1.31 [1.22, 1.42] | 4.15×10^-12^ |
| mLOY | L98 Other disorders of skin and subcutaneous tissue, not elsewhere classified | 1.2 [1.14, 1.26] | 7.50×10^-12^ |
| mLOY | B01 Varicella [chickenpox] | 0.76 [0.71, 0.83] | 9.84×10^-12^ |
| mLOY | Y83 Surgical operation and other surgical procedures as the cause of abnormal reaction of the patient, or of later complication, without mention of misadventure at the time of the procedure | 1.28 [1.19, 1.37] | 1.81×10^-11^ |
| mLOY | J18 Pneumonia, organism unspecified | 1.25 [1.17, 1.34] | 2.31×10^-11^ |
| mLOY | M35 Other systemic involvement of connective tissue | 2 [1.63, 2.46] | 3.32×10^-11^ |
| mLOY | H60 Otitis externa | 1.24 [1.16, 1.32] | 3.53×10^-11^ |
| mLOY | I63 Cerebral infarction | 1.61 [1.4, 1.86] | 7.32×10^-11^ |
| mLOY | I22 Subsequent myocardial infarction | 1.98 [1.61, 2.43] | 8.69×10^-11^ |
| mLOY | J03 Acute tonsillitis | 0.78 [0.72, 0.84] | 1.08×10^-10^ |
| mLOY | R39 Other symptoms and signs involving the urinary system | 1.4 [1.26, 1.55] | 1.73×10^-10^ |
| mLOY | B02 Zoster [herpes zoster] | 1.32 [1.21, 1.44] | 3.20×10^-10^ |
| mLOY | M48 Other spondylopathies | 1.35 [1.23, 1.48] | 4.04×10^-10^ |
| mLOY | J33 Nasal polyp | 1.31 [1.2, 1.42] | 6.69×10^-10^ |
| mLOY | Z46 Fitting and adjustment of other devices | 1.4 [1.26, 1.56] | 7.48×10^-10^ |
| mLOY | H04 Disorders of lachrymal system | 1.4 [1.26, 1.56] | 8.71×10^-10^ |
| mLOY | M06 Other rheumatoid arthritis | 1.39 [1.25, 1.54] | 9.42×10^-10^ |
| mLOY | K22 Other diseases of oesophagus | 1.27 [1.17, 1.38] | 2.74×10^-9^ |
| mLOY | I34 Nonrheumatic mitral valve disorders | 1.62 [1.38, 1.91] | 4.53×10^-9^ |
| mLOY | L70 Acne | 0.65 [0.57, 0.75] | 4.58×10^-9^ |
| mLOY | I80 Phlebitis and thrombophlebitis | 1.24 [1.15, 1.33] | 6.65×10^-9^ |
| mLOY | H34 Retinal vascular occlusions | 1.8 [1.47, 2.21] | 1.08×10^-8^ |
| mLOY | I44 Atrioventricular and left bundle-branch block | 1.56 [1.34, 1.82] | 1.16×10^-8^ |
| mLOY | K62 Other diseases of anus and rectum | 1.16 [1.1, 1.22] | 2.36×10^-8^ |
| mLOY | R04 Haemorrhage from respiratory passages | 1.41 [1.25, 1.6] | 2.51×10^-8^ |
| mLOY | H81 Disorders of vestibular function | 1.37 [1.23, 1.53] | 2.59×10^-8^ |
| mLOY | K46 Unspecified abdominal hernia | 1.57 [1.34, 1.85] | 3.95×10^-8^ |
| mLOY | I67 Other cerebrovascular diseases | 1.62 [1.36, 1.93] | 6.86×10^-8^ |
| mLOY | Z03 Medical observation and evaluation for suspected diseases and conditions | 1.24 [1.14, 1.33] | 8.94×10^-8^ |
| mLOY | K92 Other diseases of digestive system | 1.28 [1.17, 1.41] | 1.75×10^-7^ |
| mLOY | I47 Paroxysmal tachycardia | 1.4 [1.24, 1.59] | 1.85×10^-7^ |
| mLOY | K20 Oesophagitis | 1.23 [1.14, 1.33] | 2.71×10^-7^ |
| mLOY | R69 Unknown and unspecified causes of morbidity | 1.17 [1.1, 1.24] | 4.69×10^-7^ |
| mLOY | D04 Carcinoma in situ of skin | 1.79 [1.43, 2.25] | 4.77×10^-7^ |
| mLOY | J92 Pleural plaque | 1.86 [1.46, 2.37] | 5.63×10^-7^ |
| mLOY | J98 Other respiratory disorders | 1.29 [1.17, 1.43] | 9.14×10^-7^ |
| mLOY | Z88 Personal history of allergy to drugs, medicaments and biologocal substances | 1.22 [1.13, 1.33] | 1.01×10^-6^ |
| mLOY | T81 Complications of procedures, not elsewhere classified | 1.24 [1.13, 1.34] | 1.01×10^-6^ |
| mLOY | I24 Other acute ischaemic heart diseases | 1.62 [1.33, 1.96] | 1.11×10^-6^ |
| mLOY | D07 Carcinoma in situ of other and unspecified genital organs | 1.97 [1.5, 2.6] | 1.11×10^-6^ |
| mLOY | H33 Retinal detachments and breaks | 1.31 [1.17, 1.46] | 1.30×10^-6^ |
| mLOY | E11 Non-insulin-dependent diabetes mellitus | 1.15 [1.08, 1.21] | 1.69×10^-6^ |
| mLOY | J90 Pleural effusion, not elsewhere classified | 1.43 [1.24, 1.66] | 1.83×10^-6^ |
| mLOY | E87 Other disorders of fluid, electrolyte and acid-base balance | 1.38 [1.21, 1.58] | 2.05×10^-6^ |
| mLOY | K59 Other functional intestinal disorders | 1.26 [1.15, 1.39] | 2.20×10^-6^ |
| mLOY | B24 Unspecified human immunodeficiency virus [HIV] disease | 0.44 [0.31, 0.62] | 2.47×10^-6^ |
| mLOY | J84 Other interstitial pulmonary diseases | 1.74 [1.38, 2.19] | 2.96×10^-6^ |
| mLOY | I95 Hypotension | 1.41 [1.22, 1.63] | 3.15×10^-6^ |
| mLOY | E14 Unspecified diabetes mellitus | 1.11 [1.06, 1.16] | 3.38×10^-6^ |
| mLOY | R00 Abnormalities of heart beat | 1.31 [1.17, 1.47] | 3.44×10^-6^ |
| mLOY | K43 Ventral hernia | 1.35 [1.19, 1.53] | 3.69×10^-6^ |
| mLOY | H90 Conductive and sensorineural hearing loss | 1.28 [1.15, 1.43] | 4.22×10^-6^ |
| mLOY | D09 Carcinoma in situ of other and unspecified sites | 1.85 [1.42, 2.42] | 6.45×10^-6^ |
| mLOY | H02 Other disorders of eyelid | 1.26 [1.14, 1.4] | 8.79×10^-6^ |
| mLOY | E05 Thyrotoxicosis [hyperthyroidism] | 1.41 [1.21, 1.64] | 9.16×10^-6^ |
| mLOY | Z90 Acquired absence of organs, not elsewhere classified | 1.32 [1.16, 1.49] | 1.20×10^-5^ |
| mLOY | I46 Cardiac arrest | 2.02 [1.47, 2.76] | 1.22×10^-5^ |
| mLOY | K65 Peritonitis | 1.38 [1.2, 1.6] | 1.26×10^-5^ |
| mLOY | M18 Arthrosis of first carpometacarpal joint | 2.44 [1.63, 3.64] | 1.38×10^-5^ |
| mLOY | J41 Simple and mucopurulent chronic bronchitis | 5.05 [2.43, 10.5] | 1.45×10^-5^ |
| mLOY | M20 Acquired deformities of fingers and toes | 1.28 [1.14, 1.43] | 1.45×10^-5^ |
| mLOY | K01 Embedded and impacted teeth | 0.61 [0.49, 0.76] | 1.65×10^-5^ |
| mLOY | R91 Abnormal findings on diagnostic imaging of lung | 1.55 [1.27, 1.9] | 2.02×10^-5^ |
| mLOY | G43 Migraine | 0.84 [0.78, 0.91] | 2.05×10^-5^ |
| mLOY | N21 Calculus of lower urinary tract | 1.66 [1.31, 2.1] | 2.22×10^-5^ |
| mLOY | I84 Haemorrhoids | 1.14 [1.07, 1.21] | 2.74×10^-5^ |
| mLOY | I35 Nonrheumatic aortic valve disorders | 1.41 [1.2, 1.66] | 2.90×10^-5^ |
| mLOY | J61 Pneumoconiosis due to asbestos and other mineral fibres | 1.83 [1.37, 2.45] | 3.74×10^-5^ |
| mLOY | B27 Infectious mononucleosis | 0.72 [0.62, 0.84] | 3.75×10^-5^ |
| mLOY | D41 Neoplasm of uncertain or unknown behaviour of urinary organs | 1.55 [1.26, 1.92] | 3.96×10^-5^ |
| mLOY | I69 Sequelae of cerebrovascular disease | 1.65 [1.3, 2.09] | 4.16×10^-5^ |
| mLOY | H53 Visual disturbances | 1.23 [1.11, 1.36] | 4.29×10^-5^ |
| mLOY | H93 Other disorders of ear, not elsewhere classified | 1.17 [1.09, 1.27] | 4.37×10^-5^ |
| mLOY | Z60 Problems related to social environment | 1.46 [1.22, 1.75] | 4.50×10^-5^ |

**Table S8.** Statistically significant associations between medication and mCAs adjusted for age, age^2^, and smoking. No statistically significant associations were observed for autosomal mCAs or mLOX after Bonferroni correction.

| mCAs | Medication | Odds Ratio [95% Confidence interval] | P-value |
| --- | --- | --- | --- |
| mLOY | A10B blood glucose lowering drugs, excl. insulins | 0.73 [0.7-0.75] | 4.24×10^-25^ |
| mLOY | M04A antigout preparations | 0.68 [0.65-0.71] | 1.25×10^-18^ |
| mLOY | R01A decongestants and other nasal preparations for topical use | 1.1 [1.08-1.12] | 2.38×10^-8^ |
| mLOY | C08C selective calcium channel blockers with mainly vascular effects | 0.89 [0.87-0.9] | 2.42×10^-8^ |
| mLOY | S01X other ophthalmologicals in atc | 1.12 [1.1-1.14] | 1.37×10^-7^ |
| mLOY | C07A beta blocking agents | 0.91 [0.89-0.93] | 4.29×10^-6^ |
| mLOY | A11C vitamin A and D, incl. combinations of the two | 1.1 [1.08-1.12] | 7.02×10^-6^ |
| mLOY | A10A insulins and analogues | 0.78 [0.74-0.83] | 9.18×10^-6^ |
| mLOY | C10A lipid modifying agents, plain | 0.94 [0.93-0.95] | 2.17×10^-5^ |
| mLOY | C02C antiadrenergic agents, peripherally acting | 0.84 [0.8-0.88] | 1.10×10^-4^ |
| mLOY | C03C high-ceiling diuretics | 0.81 [0.77-0.86] | 1.78×10^-4^ |

**Table S9.** Associations between diseases with previously published associations with mLOY stratified by age 65.

|  | Age ≤ 65 | |  | Age > 65 | |
| --- | --- | --- | --- | --- | --- |
| Disease | Odds Ratio | P-value |  | Odds Ratio | P-value |
| C34 Malignant neoplasm of bronchus and lung | 1.01 | 0.913 |  | 0.95 | 0.555 |
| C62 Malignant neoplasm of testis | 0.87 | 0.403 |  | 0.63 | 7.45×10^-2^ |
| E06 Thyroiditis | 0.98 | 0.9.27 |  | 1.26 | 0.526 |
| G30 Alzheimer's disease | 1 | 0.984 |  | 1.02 | 0.823 |
| I71 Aortic aneurysm and dissection | 1.11 | 8.95×10^-2^ |  | 1.13 | 4.19×10^-2^ |
| K74 Fibrosis and cirrhosis of liver | 0.92 | 0.365 |  | 0.93 | 0.577 |

**Table S10.** Associations between diseases with previously published associations with mLOY stratified by cell fraction.

|  | Cell fraction > 0.03 | |  | Cell fraction > 0.1 | |  | Cell fraction > 0.2 | |
| --- | --- | --- | --- | --- | --- | --- | --- | --- |
| Disease | Odds Ratio | P-value |  | Odds Ratio | P-value |  | Odds Ratio | P-value |
| C34 Malignant neoplasm of bronchus and lung | 1.04 | 0.521 |  | 1.15 | 7.57×10^-2^ |  | 1.21 | 6.42×10^-2^ |
| C62 Malignant neoplasm of testis | 0.75 | 0.141 |  | 0.97 | 0.914 |  | 0.92 | 0.851 |
| E06 Thyroiditis | 1.23 | 0.391 |  | 1.65 | 0.109 |  | 1.67 | 0.273 |
| G30 Alzheimer's disease | 0.92 | 0.317 |  | 0.87 | 0.226 |  | 1.17 | 0.316 |
| H35 Other retinal disorders | 0.95 | 0.197 |  | 1.04 | 0.503 |  | 1 | 0.969 |
| I71 Aortic aneurysm and dissection | 1.03 | 0.505 |  | 1.18 | 9.82×10^-3^ |  | 1.28 | 4.53×10^-3^ |
| K74 Fibrosis and cirrhosis of liver | 0.88 | 0.155 |  | 0.99 | 0.904 |  | 1.08 | 0.683 |

**Table S11.** Comparison of associations between mLOY and diseases among different smoking adjustments. All models adjusted for age and age^2^ in addition to the smoking variables indicated in the table.

|  | Without smoking | |  | 3-level smoking | |  | 25-level smoking | |
| --- | --- | --- | --- | --- | --- | --- | --- | --- |
|  | Odds Ratio | P-value |  | Odds Ratio | P-value |  | Odds Ratio | P-value |
| F17 Mental and behavioural disorders due to use of tobacco | 1.7 | 5.78×10^-101^ |  | 1.08 | 3.55×10^-3^ |  | 0.99 | 0.698 |
| J44 Other chronic obstructive pulmonary disease | 1.44 | 9.43×10^-46^ |  | 1.17 | 6.71×10^-9^ |  | 1.04 | 0.162 |
| M05 Seropositive rheumatoid arthritis | 1.68 | 6.59×10^-4^ |  | 1.55 | 4.50×10^-3^ |  | 1.46 | 1.53×10^-2^ |
| M06 Other rheumatoid arthritis | 1.07 | 0.282 |  | 1.01 | 0.828 |  | 0.99 | 0.912 |
| Z59 Problems related to housing and economic circumstances | 1.97 | 3.65×10^-3^ |  | 1.66 | 3.13×10^-2^ |  | 1.62 | 4.13×10^-2^ |

**Table S12.** Comparison of associations between mLOY and diseases among different age adjustments. All models adjusted for 25-level smoking in addition to the age variables indicated in the table.

|  | Without age | |  | Linear age | |  | With quadratic age | |
| --- | --- | --- | --- | --- | --- | --- | --- | --- |
| Disease | Odds Ratio | P-value |  | Odds Ratio | P-value |  | Odds Ratio | P-value |
| F11 Mental and behavioural disorders due to use of opioids | 0.33 | 5.23×10^-3^ |  | 0.44 | 4.81×10^-2^ |  | 0.46 | 5.89×10^-2^ |
| M19 Other arthrosis | 1.28 | 2.30×10^-26^ |  | 0.95 | 4.23×10^-2^ |  | 0.96 | 9.98×10^-2^ |
| N25 Disorders resulting from impaired renal tubular function | 0.62 | 2.06×10^-1^ |  | 0.46 | 4.80×10^-2^ |  | 0.47 | 5.27×10^-2^ |
| R35 Polyuria | 1.22 | 3.39×10^-3^ |  | 0.87 | 4.44×10^-2^ |  | 0.87 | 5.17×10^-2^ |
| Z94 Transplanted organ and tissue status | 0.83 | 1.61×10^-1^ |  | 0.75 | 3.29×10^-2^ |  | 0.77 | 5.01×10^-2^ |
